# Incorporating nonlinearity into mediation analyses

**DOI:** 10.1186/s12874-017-0296-6

**Published:** 2017-03-21

**Authors:** George J. Knafl, Kathleen A. Knafl, Margaret Grey, Jane Dixon, Janet A. Deatrick, Agatha M. Gallo

**Affiliations:** 1 School of Nursing, University of North Carolina at Chapel Hill, 5014 Carrington Hall, Campus Box 7460, Chapel Hill, NC 27599-7460 USA; 2 School of Nursing, Yale University, New Haven, USA; 3 School of Nursing, University of Pennsylvania, Philadelphia, USA; 4 College of Nursing, University of Illinois at Chicago, Chicago, USA

**Keywords:** Adaptive regression, Childhood chronic conditions, Fractional polynomials, Mediation, Moderated mediation, Nonlinearity

## Abstract

**Background:**

Mediation is an important issue considered in the behavioral, medical, and social sciences. It addresses situations where the effect of a predictor variable *X* on an outcome variable *Y* is explained to some extent by an intervening, mediator variable *M*. Methods for addressing mediation have been available for some time. While these methods continue to undergo refinement, the relationships underlying mediation are commonly treated as linear in the outcome *Y*, the predictor *X*, and the mediator *M*. These relationships, however, can be nonlinear. Methods are needed for assessing when mediation relationships can be treated as linear and for estimating them when they are nonlinear.

**Methods:**

Existing adaptive regression methods based on fractional polynomials are extended here to address nonlinearity in mediation relationships, but assuming those relationships are monotonic as would be consistent with theories about directionality of such relationships.

**Results:**

Example monotonic mediation analyses are provided assessing linear and monotonic mediation of the effect of family functioning (*X*) on a child’s adaptation (*Y*) to a chronic condition by the difficulty (*M*) for the family in managing the child's condition. Example moderated monotonic mediation and simulation analyses are also presented.

**Conclusions:**

Adaptive methods provide an effective way to incorporate possibly nonlinear monotonicity into mediation relationships.

## Background

Mediation is an important issue considered in the behavioral, medical, and social sciences, addressing situations where the effect of a predictor variable *X* on an outcome (or dependent or response) variable *Y* is explained to some extent by an intervening, mediator variable *M*. Methods for addressing mediation have existed for some time [1–3]. Since then, they have undergone a variety of refinements [4–28].

Relationships underlying mediation are commonly treated as linear in *Y*, *X*, and *M*. Mediating relationships, however, can be nonlinear. A few authors have addressed nonlinearity in the mediation context [29–32]. Pearl [30, 31] has developed a general approach for quantifying direct, indirect, and total effects allowing for nonlinearity as well as for categorical variables. Standard polynomials sometimes can be effectively used to address nonlinearity in predictors, but not in general [33]. To fully address nonlinearity requires more complex methods like nonparametric regression. Methods are needed to assess linearity of mediation relationships and to conduct mediation analyses when the underlying relationships are nonlinear.

The purpose of this paper is 1. To present an approach for assessing linearity of mediation relationships and for conducting nonlinear mediation analyses, based on the adaptive regression methods of Knafl and Ding [34], and 2. To provide example analyses using these methods. Adaptive methods were originally developed for nonlinear modeling in the Poisson regression context with Poisson distributed count outcomes [35], but the methods extend to linear regression with continuous outcomes treated as normally distributed, as often used in mediation analyses, as well as logistic regression with discrete outcomes [36]. SAS® (SAS Institute, Inc., Cary, NC) macros have been developed to support adaptive regression. Knafl [37] presents examples of the use of one of these macros for nonlinear growth curve modeling. These methods address not only nonlinearity in predictors but also nonlinearity in outcomes when those outcomes are continuous and positive valued. They also allow for correlated outcomes and/or non-constant variances.

Behavioral, medical, and social science theories usually do not hypothesize that relationships are nonlinear but implicitly assume they are linear. However, Hayes and Preacher [29] provide a variety of examples of behavioral theories that incorporate nonlinearity. In any case, hypothesized relationships are usually stated in terms of directionality: as *X* increases, *Y* increases or decreases. Such statements are not inherently linear but can be represented more generally by possibly nonlinear monotonic relationships. Monotonicity is operationalized in this paper with single power transforms. Moderated monotonic mediation is also addressed in the paper.

## Methods

This section starts by formulating standard linear mediation relationships, then nonlinear mediation relationships followed by monotonic mediation relationships. Adaptive regression methods are described next along with how they can be used to assess monotonic mediation and moderated monotonic mediation. The section ends with a description of the two data sets used in example analyses.

### Linear mediation relationships

**Fig. 1:**
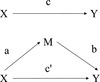
Linear mediation relationships

As commonly considered, regression models underlying mediation are formulated as linear in *Y*, *X*, and *M*. As an example, work pressure *X* could result in increased work stress *M* leading to increased alcohol consumption *Y*. The following models are considered relating *Y* to *X* (e.g., alcohol consumption to work pressure), *M* to *X* (e.g., work stress to work pressure), and *Y* to *M* and *X* (e.g., alcohol consumption to work pressure controlling for work stress), respectively (Fig. 1).

1 $$ Y = {i}_{Y,1}+ c\cdot X+{U}_{Y,1} $$

2 $$ M = {i}_M+ a\cdot X+{U}_M $$

3 $$ Y = {i}_{Y,2}+{c}^{\mathit{\hbox{'}}}\cdot X+ b\cdot M+{U}_{Y,2} $$

The random variables *U*_*Y*,1_, *U*_*M*_, and *U*_*Y*,2_ are assumed to have mean zero. They can be thought of as omitted factors (as in [31]) or as random errors.

The slope *c* for *X* in model (1) is the total effect of *X* on *Y* (e.g., of work pressure on alcohol consumption) while the slope *c* ' for *X* in model (3) is the direct effect of *X* on *Y* controlling for *M* (e.g., of work pressure on alcohol consumption controlling for work stress). The indirect effect Δ satisfies

$$ \varDelta = c-{c}^{\mathit{\hbox{'}}}= a\cdot b $$

as demonstrated by substituting model (2) for the mediator *M* into model (3), and so the total effect is the sum of the direct and indirect effects. In the Baron and Kenny [1] approach, a significant total effect *c* was considered necessary for mediation to hold. However, this no longer is considered necessary [11]. There can be a nonzero indirect effect even when the total effect is non-significant [38]. However, model (1) is often investigated in practice. In any case, a crucial issue for mediation to hold is a significantly nonzero indirect effect Δ.

A variety of tests for a zero indirect effect are available. Sobel’s test [21, 39] is the best known, but MacKinnon et al. [14] considered 14 alternatives including Sobel’s test. Difference in coefficients approaches capitalize on the first equality for Δ while product of coefficients approaches including Sobel’s test capitalize on the second equality. The assumptions underlying these tests can be questionable, and so the test based on the bootstrapped confidence interval (CI) for Δ has been proposed as a robust alternative [21, 29, 40, 41], especially when bias-corrected [42]. However, bias-corrected bootstrapped CIs can generate inflated Type I errors [43].The strength of the indirect effect [21] can be measured by the relative indirect effect $$ {\varDelta}^{\hbox{'}}=\raisebox{1ex}{$\varDelta $}\!\left/ \!\raisebox{-1ex}{$ c$}\right. $$ (often denoted as *P*_*M*_).

Since mediation addresses causality, temporal precedence is an important issue. This can be addressed by measuring *X* before measuring *M* and that before measuring *Y* [44]. However, mediation sometimes is addressed using cross-sectional data. Equations (1)-(3) are the same for these two cases. Random assignment of the settings of *X* provides support for causality for the *X* to *Y* relationship and the *X* to *M* relationship, but the *M* to *Y* relationship can still be confounded, and even when *X*, *M*, and *Y* have been measured longitudinally [45]. Nonexperimental or observational studies need theoretical justification for hypothesized causal relationships [21]. Pearl [31] has provided sufficient conditions for identifying direct, indirect, and total effects for observational studies.

### Nonlinear mediation relationships

#### General approach for addressing nonlinearity

Nonlinear mediation involves the following two regression models (4)-(5) generalizing the linear regression models (2)-(3) while no longer considering model (1).

4 $$ M = {i}_M+{T}_{M, X}(X)+{U}_M $$

5 $$ Y = {i}_Y+{T}_{Y, X}(X)+{T}_{Y, M}(M)+{U}_Y $$

*T*_*M*,*X*_(*X*) and *T*_*Y*,*X*_(*X*) are possibly nonlinear transforms of the predictor *X* while *T*_*Y,M*_(*M*) is a possibly nonlinear transform of the mediator *M*. The parameters *i*_*M*_ and *i*_*Y*_ are standard intercepts. When one of the variables *X* or *M* is discrete, only its identity transform is considered.

As an example, Hayes and Preacher [29] considered the special case with

6 $$ {T}_{M, X}( X)= a\cdot \log X,\kern0.5em {T}_{Y, X}( X)={c}^{\mathit{\hbox{'}}}\cdot X,\kern0.47em \mathrm{and}\kern0.37em {T}_{Y, M}( M)={b}_1\cdot M+{b}_2\cdot {M}^2 $$

where log denotes the natural log transform. Following Stolzenberg [32], they defined the instantaneous indirect effect, generalizing the product of coefficients approach, as the product of the derivatives $\frac{\partial M}{\partial X}$ and $\frac{\partial Y}{\partial M}$ replacing *M* by model (4) and assuming *U*_*M*_ and *U*_*Y*_ have no impact on these derivatives. For their example (6), this equals

7 $$ \begin{array}{l}\\ {}\left({b}_1+2\cdot {b}_2\cdot \left({i}_M+ a\cdot \log X\right)\right)\cdot \frac{a}{X}\end{array} $$

(as also reported in their eq. (10)).

#### Pearl’s more general approach

Pearl [31] provided the following general model for addressing nonlinearity in the mediation context, also allowing for categorical variables,

8 $$ m={f}_M\left( x,{u}_M\right)\ \mathrm{and}\  y={f}_Y\left( x, m,{u}_y\right) $$

where *x*, *m*, *y*, *u*_*M*_, and *u*_*Y*_ are possible values for the random variables *X*, *M*, *Y*, *U*_*M*_, and *U*_*Y*_ while *f*_*M*_ and *f*_*Y*_ are general transforms. For example, the values *m* of work stress *M* could depend nonlinearly on the values *x* of work pressure *X* and the values *u*_*M*_ of the omitted factor or error variable *U*_*M*_ while the values *y* of alcohol consumption *Y* could depend nonlinearly on the values *x* of work pressure *X*, the values *m* of work stress *M*, and the values *u*_*y*_ of the omitted factor or error variable *U*_*Y*_. Pearl also included the relationship *x* = *f*_*X*_(*u*_*X*_), but that is not needed in what follows. He assumed that variables have been standardized, but that is not assumed here. He provided definitions of total, natural direct, and natural indirect effects under model (8), which generalize those effects for the linear case of models (1)-(3).

The natural direct effect for a change in value of *X* from *x* to *x* ' is defined as the expected value *NDE*(*x* ', *x*) = *E*(*nde*(*x* ', *x*)) where

$$ n d e\left( x\mathit{\hbox{'}}, x\right)={f}_Y\left( x\mathit{\hbox{'}},{f}_M\left( x,{u}_M\right),{u}_Y\right)-{f}_Y\left( x,{f}_M\left( x,{u}_M\right),{u}_Y\right). $$

In other words, *nde*(*x* ', *x*) is the change in *y* when *x* changes to *x* ' while *m* is held fixed at its value for the initial value *x*. Expectations, here and in what follows, are with respect to *U*_*M*_ and *U*_*Y*_. The natural indirect effect for a change in value of *x* to *x* ' is the expected value *NIE*(*x* ', *x*) = *E*(*nie*(*x* ', *x*)) where

$$ n i e\left( x\mathit{\hbox{'}}, x\right)={f}_Y\left( x,{f}_M\left( x\mathit{\hbox{'}},{u}_M\right),{u}_Y\right)-{f}_Y\left( x,{f}_M\left( x,{u}_M\right),{u}_Y\right). $$

In other words, *nie*(*x* ', *x*) is the change in *y* when *x* is held fixed at its initial value while *m* changes from its value for *x* to its value for *x* '. The total effect for a change in value of *x* to *x* ' is the expected value *TE*(*x* ', *x*) = *E*(*te*(*x* ', *x*)) where

$$ t e\left( x\mathit{\hbox{'}}, x\right)={f}_Y\left( x\mathit{\hbox{'}},{f}_M\left( x\mathit{\hbox{'}},{u}_M\right),{u}_Y\right)-{f}_Y\left( x,{f}_M\left( x,{u}_M\right),{u}_Y\right). $$

In other words, *te*(*x* ', *x*) is the change in *y* when *x* changes to *x* ' and *m* changes from its value for *x* to its value for *x* '.

Adding and subtracting *f*_*Y*_(*x* ', *f*_*M*_(*x*, *u*_*M*_), *u*_*Y*_) to *te*(*x* ', *x*) gives that

$$ T E\left( x\mathit{\hbox{'}}, x\right)= N D E\left( x\mathit{\hbox{'}}, x\right)- N I E\left( x, x\mathit{\hbox{'}}\right) $$

(also eq. (14) of [31]). Thus, the total effect is only the sum of the natural direct and natural indirect effects in the special case that *NIE*(*x* ', *x*) = − *NIE*(*x*, *x* '), which holds for the linear mediation case of models (1)-(3). This result indicates the shortcoming of trying to generalize the indirect effect using a difference of coefficients approach.

The definitions of *NDE*(*x* ', *x*), *NIE*(*x* ', *x*), and *TE*(*x* ', *x*) are sufficient for handling categorical predictors *X*. For the case of continuous predictors *X*, they can be used to define instantaneous natural direct, natural indirect, and total effects using limits as follows:

$$ \frac{dNDE(x)}{dx}=\underset{x\hbox{'}\to x}{ \lim}\frac{NDE\left( x\mathit{\hbox{'}}, x\right)}{x\mathit{\hbox{'}}- x}, $$

$$ \frac{dNIE(x)}{dx}=\underset{x\hbox{'}\to x}{ \lim}\frac{NIE\left( x\mathit{\hbox{'}}, x\right)}{x\mathit{\hbox{'}}- x}, $$

and

$$ \frac{dTE(x)}{dx}=\underset{x\hbox{'}\to x}{ \lim}\frac{TE\left( x\mathit{\hbox{'}}, x\right)}{x\mathit{\hbox{'}}- x}. $$

The relative instantaneous natural indirect effect function can be computed as

$$ \Delta \mathit{\hbox{'}}=\raisebox{1ex}{$\frac{dNIE(x)}{dx}$}\!\left/ \!\raisebox{-1ex}{$\frac{dTE(x)}{dx}.$}\right. $$

#### Examples

For the Hayes and Preacher [29] nonlinear example, *m* = *f*_*M*_(*x*, *u*_*M*_) and *y* = *f*_*Y*_(*x*, *m*, *u*_*Y*_) are determined by eqs. (4)-(5) evaluated at (6). Thus,

$$ n d e\left( x\mathit{\hbox{'}}, x\right)= c\mathit{\hbox{'}}\cdot \left( x\mathit{\hbox{'}}- x\right)= N D E\left( x\mathit{\hbox{'}}, x\right) $$

so that $\frac{dNDE(x)}{dx}= c\mathit{\hbox{'}}$ as would be expected for a linear model for *Y* in *X*. Also,

$$ \begin{array}{c} nie\left( x\mathit{\hbox{'}}, x\right)={b}_1\cdot {f}_M\left( x\mathit{\hbox{'}},{u}_M\right)+{b}_2\cdot {f}_M^2\left( x\mathit{\hbox{'}},{u}_M\right)-{b}_1\cdot {f}_M\left( x,{u}_M\right)-{b}_2\cdot {f}_M^2\left( x,{u}_M\right)\\ {}={b}_1\cdot a\cdot \left( \log x\mathit{\hbox{'}}- \log x\right)+{b}_2\cdot \left({\left({i}_M+ a\cdot \log x\mathit{\hbox{'}}+{u}_M\right)}^2-{\left({i}_M+ a\cdot \log x+{u}_M\right)}^2\right)\\ {} = {b}_1\cdot a\cdot \left( \log x\mathit{\hbox{'}}- \log x\right)+{b}_2\cdot \left(2\cdot \left({i}_M+{u}_M\right) + a\cdot \left( \log x\mathit{\hbox{'}}+ \log x\right)\right)\cdot a\cdot \left( \log x\mathit{\hbox{'}}- \log x\right)\end{array} $$

so that

$$ \frac{dNIE(x)}{dx}=\left({b}_1+2\cdot {b}_2\cdot \left({i}_M+ a\cdot \log x\right)\right)\cdot \frac{a}{x}, $$

which agrees with result (7).

As a second example, consider the following case with *M* = *X* + *U*_*M*_ (i.e., the linear model (2) with *i*_*m*_ = 0 and *a* = 1) and *Y* = *i*_*Y*_ + *c* ' ⋅ *X* + *b* ⋅ *M* ^3^ + *U*_*Y*_. Since $\frac{\partial M}{\partial X}=1$ and $\frac{\partial Y}{\partial M}= b\cdot 3\cdot {M}^2,$ the instantaneous indirect effect for a fixed value *x* of *X* using the product of coefficients approach of Hayes and Preacher [29] would be *b* ⋅ 3 ⋅ *x* ^2^ (i.e., substituting *x* for *M* based on the relationship *M* = *X* + *U*_*M*_ and ignoring the associated value *u*_*M*_ for *U*_*M*_). On the other hand, the general approach of Pearl [31] gives

$$ n i e\left( x\mathit{\hbox{'}}, x\right)= b\cdot {f}_M^3\left( x\mathit{\hbox{'}},{u}_M\right)- b\cdot {f}_M^3\left( x,{u}_M\right)= b\cdot \left({\left( x\mathit{\hbox{'}}+{u}_M\right)}^3-{\left( x+{u}_M\right)}^3\right) $$

so that

$$ \underset{x\mathit{\hbox{'}}\to x}{ \lim}\frac{nie\left( x\mathit{\hbox{'}}, x\right)}{x\mathit{\hbox{'}} - x}= b\cdot 3\cdot {\left( x+{u}_M\right)}^2= b\cdot 3\cdot \left({x}^2+2\cdot x\cdot {u}_M+{u}_M^2\right). $$

Since *E*(*u*_*M*_) = 0, the instantaneous natural indirect effect satisfies

$$ \frac{dNIE(x)}{dx}= b\cdot 3\cdot \left({x}^2+ E\left({U}_M^2\right)\right). $$

The expected value *E*(*U*_*M*_^2^) > 0 except in trivial cases. Consequently, this is not the same value as obtained by the Hayes-Preacher product of coefficients approach. The problem with that approach is that *U*_*M*_ cannot always be ignored in computing indirect effects. Similar problems could occur if *f*_*Y*_ is nonlinear in *u*_*Y*_. This result indicates the shortcoming of trying to generalize the indirect effect using a product of coefficients approach.

### Monotonic mediation

Theorized relationships are reasonably operationalized as monotonic relationships. Monotonic mediation can be formulated with models (9)-(10).

9 $$ {M}^q={i}_M+ a\cdot {X}^{q\mathit{\hbox{'}}}+{U}_M $$

10 $$ {Y}^p={i}_Y+ c\mathit{\hbox{'}}\cdot {X}^{p\mathit{\hbox{'}}}+ b\cdot {M}^{q\cdot p\mathit{\hbox{'}}\mathit{\hbox{'}}}+{U}_Y $$

For example, transformed work stress *M* ^*q*^ could depend nonlinearly on transformed work pressure *X* ^*q* '^ in model (9) while transformed alcohol consumption *Y* ^*p*^ could depend nonlinearly on transformed work pressure *X* ^*p* '^ controlling for transformed work stress *M* ^*q* ⋅ *p*"^ in model (10). When Y > 0, M > 0, and X > 0, the power transforms *Y* ^*p*^, *X* ^*p* '^, *X* ^*q*^ ', *M* ^*q*^, and *M* ^*q*"^ are well-defined for arbitrary real valued powers *p*, *p* ', *q*, *q* ', and *q*" = *q* ⋅ *p* ". An approach for extending this to arbitrary valued variables is presented later. The power *p* = 0 represents the natural log transform rather than the constant transform. These are Box-Tidwell transformations [46], although Box and Tidwell considered them only for predictors. Models (9)-(10) provide for transformation of outcomes as well as predictors as opposed to just predictors as in models (4)-(5) and (8). Fractional polynomials of degree 1 in *X*, *M*, and *Y* have been used in (9)-(10) to guarantee that relationships are monotonic.

Models (9)-(10) can be represented in the general form of model (8) replacing *m* and *y* by *m* ^*q*^ and *y* ^*p*^ giving

$$ {m}^q={f}_M\left( x,{u}_M\right)={i}_M+ a\cdot {x}^{q\hbox{'}}+{u}_M $$

and

$$ {y}^p={f}_Y\left( x, m,{u}_Y\right)={i}_Y+ c\mathit{\hbox{'}}\bullet {x}^{p\mathit{\hbox{'}}}+ b\cdot {m}^{q\cdot p\mathit{\hbox{'}}\mathit{\hbox{'}}}+{u}_M. $$

Hence, *nde*(*x* ', *x*) = *c* ' ⋅ ((*x* ')^*p* '^ − *x* ^*p*^) = *NDE*(*x* ', *x*) so that the instantaneous natural direct effect satisfies

$$ \frac{dNDE(x)}{dx}= c\mathit{\hbox{'}}\cdot p\mathit{\hbox{'}}\cdot {x}^{p^{\hbox{'}}-1} $$

for *p'* ≠ 0 while $\frac{dNDE(x)}{dx}= c\mathit{\hbox{'}}\cdot {x}^{-1}$ for *p'* = 0. Also,

$$ \begin{array}{c} nie\left( x\mathit{\hbox{'}}, x\right)= b\cdot {f}_M^{p\hbox{'}\hbox{'}}\left( x\mathit{\hbox{'}},{u}_M\right)- b\cdot {f}_M^{p\hbox{'}\hbox{'}}\left( x,{u}_M\right)\\ {}= b\cdot \left({\left({i}_M+ a\cdot {\left( x\mathit{\hbox{'}}\right)}^{q^{\hbox{'}}}+{u}_M\right)}^{p^{\hbox{'}\hbox{'}}}-{\left({i}_M+ a\cdot {(x)}^{q\mathit{\hbox{'}}}+{u}_M\right)}^{p^{\hbox{'}\hbox{'}}}\right).\end{array} $$

Note that *nie*(*x*, *x* ') = − *nie*(*x* ', *x*) so that *te*(*x* ', *x*) = *nde*(*x* ', *x*) + *nie*(*x* ', *x*), and so the total effect is the sum of the natural direct and natural indirect effects as for the linear mediation case of models (1)-(3).

#### Examples

**Fig. 2:**
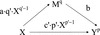
Monotonic mediation relationships (assuming $q^{\prime}\ne 0$ and $p^{\prime}\ne 0$).

For the special case with *p*'' = 1,

$$ n i e\left( x\mathit{\hbox{'}}, x\right)= b\cdot a\cdot \left({\left( x\mathit{\hbox{'}}\right)}^{q\mathit{\hbox{'}}}-{x}^{q\mathit{\hbox{'}}}\right)= N I E\left( x\mathit{\hbox{'}}, x\right) $$

so that

$$ \frac{dNIE(x)}{dx}= b\cdot a\cdot q\mathit{\hbox{'}}\cdot {x}^{q\mathit{\hbox{'}}-1} $$

for *q* ' ≠ 0 while $\frac{dNIE(x)}{dx}= b\cdot a\cdot {x}^{-1}$ for *q* ' = 0. Figure 2 represents this model (assuming *q* ' ≠ 0 and *p* ' ≠ 0). The paths are labeled with derivatives of expected values for *M* ^*q*^ and *Y* ^*p*^ relative to *X* or to *M* ^*q*^ generalizing the slopes used in Figure 1. Note that as in the linear case of Figure 1, the instantaneous natural indirect effect equals the product *b* ⋅ *a* ⋅ *q* ' ⋅ *x* ^*q* ' − 1^ of the labels for the two upper paths and the instantaneous natural direct effect equals the label *c* ' ⋅ *p* ' ⋅ *x* ^*p* ' − 1^ for the lower path.

For the special case with *p*" = 2 and *q* " ≠ 0,

$$ \begin{array}{c} nie\left( x\mathit{\hbox{'}}, x\right)= b\cdot \left({\left({i}_M+ a\cdot {\left( x\mathit{\hbox{'}}\right)}^{q\mathit{\hbox{'}}}+{u}_M\right)}^2-{\left({i}_M+ a\cdot {x}^{q\mathit{\hbox{'}}}+{u}_M\right)}^2\right)\\ {}= b\cdot \left(2\cdot \left({i}_M+{u}_M\right)+ a\cdot {\left( x\mathit{\hbox{'}}\right)}^{q\mathit{\hbox{'}}}+ a\cdot {x}^{q\mathit{\hbox{'}}}\right)\cdot a\cdot \left({\left( x\mathit{\hbox{'}}\right)}^{q\mathit{\hbox{'}}}-{x}^{q\mathit{\hbox{'}}}\right)\end{array} $$

so that

$$ \frac{dNIE(x)}{dx}= b\cdot 2\cdot \left({i}_M+ a\cdot {x}^{q\mathit{\hbox{'}}}\right)\cdot a\cdot q\mathit{\hbox{'}}\cdot {x}^{q\mathit{\hbox{'}}-1}. $$

For the special case with *p*" = 3 and *q* ≠ 0,

$$ n i e\left( x\mathit{\hbox{'}}, x\right)= b\cdot \left({\left({i}_M+ a\cdot {\left( x\mathit{\hbox{'}}\right)}^{q\mathit{\hbox{'}}}+{u}_M\right)}^3-{\left({i}_M+ a\cdot {x}^{q\mathit{\hbox{'}}}+{u}_M\right)}^3\right) $$

so that

$$ \begin{array}{c}\underset{x\hbox{'}\to x}{ \lim}\frac{nie\left( x\mathit{\hbox{'}}, x\right)}{x\mathit{\hbox{'}} - x}= b\cdot 3\cdot {\left({i}_M+ a\cdot {x}^{q\mathit{\hbox{'}}}+{u}_M\right)}^2\cdot a\cdot q\mathit{\hbox{'}}\cdot {x}^{q\mathit{\hbox{'}}-1}\\ {}= b\cdot 3\cdot \left({\left({i}_M+ a\cdot {x}^{q\mathit{\hbox{'}}}\right)}^2+2\cdot \left({i}_M+ a\cdot {x}^{q\mathit{\hbox{'}}}\right)\cdot {u}_M+{u}_M^2\right)\cdot a\cdot q\mathit{\hbox{'}}\cdot {x}^{q\mathit{\hbox{'}}-1}.\end{array} $$

Hence, the instantaneous natural indirect effect satisfies

$$ \frac{dNIE(x)}{dx}= b\cdot 3\cdot \left({\left({i}_M+ a\cdot {x}^{q\mathit{\hbox{'}}}\right)}^2+\mathrm{E}\left({U}_M^2\right)\right)\cdot a\cdot q\mathit{\hbox{'}}\cdot {x}^{q\mathit{\hbox{'}}-1}. $$

We recommend using the special case with *p* " = 1 due to its desirable properties. It provides a natural generalization of the linear case of models (1)-(3) to account for monotonicity. In what follows, *p* " = 1 is assumed unless otherwise stated.

### Adaptive regression

Methods are needed for estimating the relationships of models (4)-(5) and (9)-(10) and for assessing whether those relationships are non-constant in *X* or *M* and whether they are reasonably treated as linear in *Y*, *X*, and/or *M* or are distinctly nonlinear in any of those variables. We use adaptive regression modeling [34] for these purposes. Methods are needed as well for assessing whether the instantaneous natural indirect effect $\frac{dNIE(x)}{dx}$ is nonzero. This can be addressed as in linear mediation with bootstrapped CIs, but now computed for a grid of possible values *x* for *X*.

Knafl et al. [35] developed adaptive regression methods for nonlinear modeling of Poisson distributed count outcome variables *Y*. Knafl et al. [36] extended this to generalized linear modeling including adaptive linear regression with outcomes treated as normally distributed, as often used in mediation analyses (but they used these methods to address modeling of electronically monitored medication adherence data rather than mediation). How adaptive regression analyses are conducted is described next.

Adaptive regression methods use likelihood cross-validation (LCV) scores (as defined later) to evaluate and compare models. These scores generalize to contexts where estimation is based on maximizing likelihood-like functions such as extended quasi-likelihood functions [47]. Heuristic (i.e., rule-based) search techniques guided by LCV scores are used to identify effective fractional polynomial models [33] in primary predictor variables including predictors$X$ and mediators *M* as needed for mediation. Fractional polynomials generalize standard polynomials, which use only nonnegative integer powers, to allow for the possibility of negative and fractional powers. Fractional polynomial models for continuous outcomes are linear in associated parameter vectors (consisting of the intercept and slopes for power transforms of predictors), and so they are linear models estimated using linear regression methods. However, these models are based on relationships that are in general nonlinear (or curvilinear) in the predictor *X* for models (4)-(5) and (9)-(10) as well as in the mediator *M* for models (5) and (10).

Power transforms *f*(*X*, *p*) are defined for arbitrary real valued primary predictors *X* and powers *p* by setting *f*(*X*, *p*) to *X* ^*p*^ when *X* > 0, to 0 when *X* = 0, and to cos(*π* ⋅ *p*) ⋅ |*X*|^*p*^ when *X* < 0 where$\cos$ denotes the standard cosine function, *π* is the usual constant, and |*X*| denotes the absolute value of *X*. Note that for *X* < 0, the sign of *f*(*X*, *p*) oscillates between ±1 as the power $p$ varies. For simplicity of notation, *f*(*X*, *p*) is denoted as *X* ^*p*^.

#### Likelihood cross-validation

LCV scores are computed by randomly partitioning observations into *k* disjoint sets called folds, calculating likelihoods for folds using parameter estimates computed from the remaining data in the complement of the fold, and combining these deleted likelihoods into a geometric mean deleted likelihood score. Formally, let *S* = {*s* : 1 ≤ *s* ≤ *n*} denote the index set for the $n$ subjects (or observations or cases) in the data set under analysis, ***θ*** the vector of model parameters, and *L*(⋅; ***θ***) a likelihood function or a likelihood-like function (e.g., the extended quasi-likelihood function used with generalized linear models [47]). Randomly partition *S* into *k* > 1 disjoint nonempty subsets *S*(*h*), called folds, for *h* ∈ *H* = {*h* : 1 ≤ *h* ≤ *k*}. The LCV score is defined as

$$ \mathrm{L}\mathrm{C}\mathrm{V}={\displaystyle \prod_{h\in H}}{L}^{\frac{1}{n}}\left( S(h);\boldsymbol{\theta} \Big( S\backslash S(h)\right) $$

where *L*(*S*(*h*); ***θ***(*S*\*S*(*h*))) denotes the joint likelihood for the observations with indexes in *S*(*h*) using the maximum likelihood estimate ***θ***(*S*\*S*(*h*)) of the parameter vector ***θ*** computed using the data in the complement *S*\*S*(*h*) of the fold *S*(*h*). For a given data set, the same random fold assignment is used for all models so that the LCV scores for those models are comparable. LCV scores for multivariate data are normalized by the number of outcome measurements for all subjects rather than by the number of subjects.

Larger LCV scores indicate better models, but not necessarily substantially better models. This issue is assessed with LCV ratio tests, analogous to likelihood ratio tests in being based on the χ^2^ distribution. LCV ratio tests are expressed in terms of a cutoff for a substantial (or distinct or significant) percent decrease in the LCV score, changing with the sample size. The formula for the cutoff is provided in Section 4.4.2 of Knafl and Ding [34] and in eq. (6) of Knafl et al. [36]. LCV ratio tests are more conservative than standard tests for zero coefficients (examples are provided in [48–50]) in the sense that removal from the model of a predictor with a significant slope does not always generate a substantial percent decrease in the LCV score. Consequently, LCV ratio tests are similar in effect to adjustments for multiple comparisons.

As an example, suppose that a model${M}_1$ generates a score $\mathrm{L}\mathrm{C}\mathrm{V}\left({M}_1\right)$ smaller than the score $\mathrm{L}\mathrm{C}\mathrm{V}\left({M}_2\right)$ for another model ${M}_2$. If the percent decrease in these LCV scores, that is,

$$ \frac{\mathrm{LCV}\left({M}_2\right)-\mathrm{LCV}\left({M}_1\right)}{\mathrm{LCV}\left({M}_2\right)}\cdot 100\%, $$

is larger than the cutoff for a substantial percent decrease, then model *M*_2_ substantially improves on model *M*_1_. On the other hand, if the percent decrease is less than or equal to the cutoff, model *M*_1_ is a competitive alternative to model *M*_2_. If model *M*_1_ is also simpler (e.g., based on fewer parameters or containing no versus some interactions) than model *M*_2_, then it would be preferable to model *M*_2_ as a parsimonious, competitive alternative.

#### Overview of the adaptive modeling process for a fixed outcome

Adaptive fractional polynomial models for a fixed outcome in terms of primary predictors are identified using a heuristic search process beginning with an expansion phase, systematically adding power transforms of those primary predictors into the model, followed by a contraction phase, systematically removing any extraneous power transforms and adjusting the powers of the remaining transforms to improve the LCV score. The search process is controlled by tolerance parameters indicating the change in LCV scores that can be tolerated for each step in the search process. For example, the contraction stopping tolerance parameter setting is based on a LCV ratio test so that the final selected model is parsimonious. The tolerance parameter settings change with the number of measurements in the sample, thereby adjusting the search process by the sample size. The full adaptive modeling process is formulated in Chapter 20 of [34]. That full process is needed for estimating models (4)-(5) with arbitrary nonlinearity. Estimation of the monotonic models of (9)-(10) requires a simpler search process as addressed later.

The adaptive modeling process can generate adaptive models for variances (or more generally dispersions [47]) as well as for means. For example, when *M* and/or *Y* are continuous, the omitted factors or errors *U*_*M*_ and *U*_*Y*_ can be treated as normally distributed (or at least approximately so) with non-constant variances that are functions of *X*, *M*, and/or some other primary predictors. The log of the variances is modeled as linear in the coefficient parameters for possibly power transformed primary predictors. Coefficient parameters for the means and variances are estimated jointly using maximum likelihood with likelihoods based on the normal distribution. For correlated continuous outcomes, for example, outcomes measured over clusters such as family members or patients of the same provider, likelihoods are based on the multivariate normal distribution.

Adaptive nonlinear moderation can be addressed simply by including interactions as primary predictors. More generally, the adaptive modeling process can automatically generate geometric combinations of two or more primary predictors, that is, products of power transforms of distinct subsets of the primary predictors using possibly different powers to transform those primary predictors. The subset of transforms in the geometric combinations and their powers are generated adaptively.

#### Searching for power transforms

A base model *M*_0_ is expanded to include a transform of a predictor *X* as follows. Let *M*_0_(*p*) denote the model *M*_0_ with the power transform *X* ^*p*^ added to it. A grid search is conducted first to maximize LCV(*M*_0_(*p*)) over an initial set of powers. By default, the initial powers *p* = −3, −2.5, ⋯, −0.5, 0.5, ⋯, 2.5, 3 are used, but this set can be adjusted if desired. The power 0 is purposely not considered. For the case with *X* > 0, the effect of *p* = 0 on *M*_0_ depends on whether or not *M*_0_ includes an intercept. When there is an intercept, the power 0 corresponds to the natural log transform (demonstrated by taking the limit as *p* → 0); otherwise it corresponds to the constant transform adding in an intercept parameter to *M*_0_. When *X* has zero or negative values, the effect is more complex. Not considering the power 0 avoids slowing the adaptive modeling process to check *M*_0_ to see what the effect of that power is. In any case, powers *p* close to 0 approximate the appropriate case without having to check to see which one it is. By default, the smallest powers in absolute value that are considered are ±0.0001, but this can be adjusted.

Let *p*_0_ denote the power which maximizes LCV(*M*_0_(*p*)) for this initial set of powers. When *p*_0_ = −3, a search is conducted over powers *p* = *p*_0_ + *i* ⋅ *δ* for integer multiples *i* of *δ* = −1 until

11 $$ \mathrm{L}\mathrm{C}\mathrm{V}\left({M}_0\left({p}_0+\left( i+1\right)\cdot \delta \right)\right)<\mathrm{L}\mathrm{C}\mathrm{V}\left({M}_0\left({p}_0+ i\cdot \delta \right)\right)>\mathrm{L}\mathrm{C}\mathrm{V}\left({M}_0\left({p}_0+\left( i-1\right)\cdot \delta \right)\right) $$

When *p*_0_ = 3, *δ* is set to +1 instead. These produce powers *p*_1_ = *p*_0_ + *i* ⋅ *δ* with *i* ≥ 0 for these two cases. For −3 < *p*_0_ < 3, *p*_1_ = *p*_0_. The choice of an initial power for *X* is given by *p*_1_.

Next the choice of a power *p*_2_ for changes in powers of *δ* = ±0.1 is identified through a similar search on either side of *p*_1_. Then, this is iterated searching around *p*_2_ over changes *δ* = ±0.01, then *δ* = ±0.001, and so on. By default, changes of at most *δ* = ±0.0001 are considered, but this can be adjusted. At any stage of this process, if the smallest of the three LCV scores analogous to those of inequality (11) compared to the largest of those three scores generates a percent decrease greater than the associated tolerance parameter (indicating these three LCV scores are not close to each other), continue the search with one more decimal digit; otherwise stop the process. When the process stops at the *j* ^th^ stage, the selected model is *M*_0_(*p*_*j*_).

By default, the expansion stopping tolerance is set generously to 2.5 times the cutoff for a LCV ratio test, and so it is likely a transform of *X* would be added to the base model. However, it is also possible that the expansion might not add a transform of *X* to the base model, supporting the conclusion that *X* does not have an effect on the outcome *Y* after controlling for the transforms of the base model.

Identification of a transform of a predictor to add to a base model is a small part of the full adaptive modeling process. Multiple predictors need to be considered as part of the expansion; the best transform to remove from a base model needs to be determined as part of the contraction. With each such removal, the powers of the remaining transforms need to be adjusted to improve the LCV score. However, models (9)-(10) with *p* " = 1 and *p* and *q* fixed require identification of only the single power *q* ' for *X* in (9) and the single power *p* ' for *X* in (10). An algorithm for identifying choices for these two powers is defined later.

For the general adaptive modeling process, tests for zero coefficients for transforms in selected models are inappropriate to conduct since these tests are usually significant. Due to the contraction heuristics, the removal of each transform of the selected model generates a substantial percent decrease in the LCV score. However, this is not the case for models (9)-(10) based on single transforms of *X*. While the selected power transform for *X* in one of these models generates an optimal LCV score, the LCV score for the associated constant base model might not generate a substantial percent decrease in the LCV score, and so the slope for that selected power transform might not always be significantly nonzero. However, a LCV ratio test can also be used to assess the impact of including *X* in the model versus not including it and is likely to be more conservative than the test for a zero slope for *X*.

#### Setting the number of folds

Knafl et al. [35] used$10$ folds for estimating nonlinear individual-subject medication adherence curves on the recommendation of Kohavi [51]. However, the data sets they used had limited sample sizes at most $100.$ The choice of the number *k* of folds may need to be adjusted for different sample sizes. Knafl and Grey [52] investigated this issue for exploratory factor analysis models. They found that for three different sets of items the same number of factors was selected by maximizing LCV scores as long as the value of *k* was not too small. Consequently, they recommended using the first local maximum in *k* over multiples of 5 folds for some benchmark analysis, in their case the selection of the number of factors extracted through maximum likelihood. This choice balances the need for a sufficiently large number of folds while limiting the amount of computation. Section 2.8 of [34] provides a more complete assessment of this issue.

#### The composite mediation model

Models (9)-(10) with *p* " = 1 can be combined into a single bivariate outcome model as follows. With superscript *T* denoting the transpose operator, let ***Y*** ' = (*M* ^*q*^ *Y* ^*p*^)^*T*^, ***I***_1_ = (1 0)^*T*^, ***I***_2_ = (0 1)^*T*^, ***X***_1_ = (*X* 0)^*T*^, ***X***_2_ = (0 *X*)^*T*^, ***M*** = (0 *M*)^*T*^, and ***U*** = (***U***_***M***_ ***U***_***Y***_)^***T***^. The model

12 $$ {\boldsymbol{Y}}^{\hbox{'}}={\beta}_1\cdot {\boldsymbol{I}}_1+{\beta}_2\cdot {\boldsymbol{I}}_2+{\beta}_3\cdot {\boldsymbol{X}}_1^{q\hbox{'}}+{\beta}_4\cdot {\boldsymbol{X}}_2^{p\hbox{'}}+{\beta}_5\cdot {\boldsymbol{M}}^q+\boldsymbol{U}, $$

where a power transform of a vector is the vector of its entries transformed by that power, satisfies *β*_1_ = *i*_*M*_, *β*_2_ = *i*_*Y*_, *β*_3_ = *a*, *β*_4_ = *c* ', and *β*_5_ = *b*, and so provides a single model for the five coefficient parameters for the means of models (9)-(10). Assume that ***U*** is bivariate normally distributed with covariance matrix **Σ**, thereby allowing for possibly correlated omitted factors or errors. Model (12) is a path model nonlinear in the outcome *Y*, the mediator *M*, and the predictor *X* with *Y* and *M* measured with error and *X* measured without error.

Let ***θ*** be the vector of model parameters, including *β*_*j*_ 1 ≤ *j* ≤ 5 and all the parameters for modeling the covariance matrix **Σ**. Using subscripts *s* for *s* ∈ *S* as defined earlier, the likelihood *L*(*S*; ***θ***) satisfies $\log L\left( S;\boldsymbol{\theta} \right)={\displaystyle \prod_{s\in S}}{\ell}_s$ where

$$ {\ell}_s=-\raisebox{1ex}{$1$}\!\left/ \!\raisebox{-1ex}{$2$}\right.\cdot {\boldsymbol{u}}_s^T\cdot {\boldsymbol{\varSigma}}_s^{-1}\cdot {\boldsymbol{u}}_s-\raisebox{1ex}{$1$}\!\left/ \!\raisebox{-1ex}{$2$}\right.\cdot \log \left(\left|{\boldsymbol{\varSigma}}_s\right|\right)-\raisebox{1ex}{$1$}\!\left/ \!\raisebox{-1ex}{$2$}\right.\cdot 2\cdot \log \left(2\cdot \pi \right), $$

$$ {\boldsymbol{\mu}}_s={\beta}_1\cdot {\boldsymbol{I}}_1+{\beta}_2\cdot {\boldsymbol{I}}_2+{\beta}_3\cdot {\boldsymbol{X}}_{1, s}^{q^{\hbox{'}}}+{\beta}_4\cdot {\boldsymbol{X}}_{2, s}^{p^{\hbox{'}}}+{\beta}_5\cdot {\boldsymbol{M}}_s^q, $$

**Σ**_*s*_ is the covariance matrix for the *s* ^th^ observation, |**Σ**_*s*_| its determinant, and ***u***_***s***_ = ***Y*** ' _***s***_ − ***μ***_***s***_ the associated residual vector. For model (12), there is only one correlation parameter *ρ* that is the same for all *s*, but each of the variances for *U*_*M,s*_ and for *U*_*Y,s*_ might change with *s* or be the same for all *s*.

When the omitted factors or errors are independent, that is, when *ρ* = 0,

$$ L\left( S;\boldsymbol{\theta} \right)={L}_M\left( S;\boldsymbol{\theta} \right)\cdot {L}_Y\left( S;\boldsymbol{\theta} \right) $$

separates into two terms corresponding to the likelihoods *L*_*M*_(*S*; *θ*) and *L*_*Y*_(*S*; *θ*) for models (9) and (10), respectively. The LCV score for model (12) also separates, but into

$$ \mathrm{L}\mathrm{C}\mathrm{V}={\mathrm{LCV}}_M^{\raisebox{1ex}{$1$}\!\left/ \!\raisebox{-1ex}{$2$}\right.}\cdot {\mathrm{LCV}}_Y^{\raisebox{1ex}{$1$}\!\left/ \!\raisebox{-1ex}{$2$}\right.} $$

where LCV_*M*_ and LCV_*Y*_ are LCV scores for models (9) and (10), respectively. This holds because the LCV score for model (12) is normalized by the number 2 ⋅ *n* of outcome measurements while LCV_*M*_ and LCV_*Y*_ each are normalized by the number *n* of subjects. If *q* is fixed (e.g., with *q* = 1 so models (9)-(10) are linear in *M*), maximum likelihood estimation and adaptive modeling of models (9) and (10) separately generate the same results as for modeling them in combination using model (12), assuming consistent fold assignments for these two cases. However, this only holds when *q* is fixed and *ρ* = 0. Even when *ρ* = 0, identification of an appropriate value for *q* requires comparing LCV scores for the composite model (12) since the same value of *q* is used in its submodels (9)-(10).

#### Identifying power transforms for $\mathrm{X}$ in the composite model

For fixed choices for the powers *p* and *q*, the adaptive expansion process is constrained to generate a single transform for ***X***_1_ and for ***X***_2_ as follows. Let *M*_0_ be the base model with means based only on ***I***_1_ and ***I***_2_ along with a fixed choice for the covariance matrix of *U*_*M*_ and *U*_*Y*_. Let *M*_0_(*q* ' _1_) be this base model expanded to include a power transform of ***X***_1_ with selected power *q* ' _1_ and *M*_0_(*p* ' _1_) expanded to include a power transform of ***X***_2_ with selected power *p* '_1_. If

$$ \mathrm{L}\mathrm{C}\mathrm{V}\left({M}_0\left( p{\mathit{\hbox{'}}}_1\right)\right)>\mathrm{L}\mathrm{C}\mathrm{V}\left({M}_0\left( q{\mathit{\hbox{'}}}_1\right)\right), $$

then expand *M*_0_(*p* ' _1_) to include a power transform of ***X***_1_ with power *q* ' _2_, possibly different than *q* ' _1_. Otherwise, expand *M*_0_(*q* ' _1_) to include a power transform of ***X***_2_ with power *p* ' _2_, possibly different than *p* ' _1_. This is the standard adaptive expansion process constrained to include a single power transform of primary predictors ***X***_1_ and ***X***_2_ for the means.

Since power transforms are added to the model without adjusting the powers of previously added transforms, there might be an improvement if these powers are adjusted. The formal power transform process is defined in Section 20.4.4 of [34]. Informally, each power is adjusted using the power adjustment process described earlier but starting at its current value holding the other powers fixed. Stop if no adjusted powers provide an improvement in the LCV score. Otherwise, continue the process using the adjusted power generating the best improvement in the LCV score. However, since the generated model (12) for fixed *p* and *q* has only two transforms, the improvement if any is unlikely to be substantial. Consequently, power adjustment is treated as an optional feature of the adaptive monotonic mediation process.

The model for the variances (technically, the model for the log of the variances) based on ***I***_1_ and ***I***_2_ results in separate but constant variances for *U*_*M*_ and *U*_*Y*_. The associated covariance matrix also allowing for the single general correlation *ρ* is called compound symmetry heterogeneous (CSH). Non-constant variances can be generated by considering ***X***_1_, ***X***_2_, and/or ***M*** as primary predictors for modeling the variances. The full adaptive modeling process can be used for modeling the variances since these need not be monotonic. This is achieved by including ***I***_1_ and ***I***_2_ in the base model for the variances, not restricting the expansion of the model for the variances, and restricting the contraction to contract only transforms from the model for the variances. By default, the contraction considers removal of the intercepts corresponding to ***I***_1_ and ***I***_2_ in the model for the variances, but this can be overridden.

Covariates can be included in model (12). For each covariate *Z*, include ***Z***_1_ = (*Z* 0)^*T*^ and ***Z***_2_ = (0 *Z*)^*T*^ as primary predictors for the means and/or variances, respectively, addressing models (9)-(10). The full adaptive modeling process can be used to generate power transforms of ***Z***_1_ and ***Z***_2_ for modeling the means and/or variances.

#### Power-adjusted likelihood cross-validation

Assume that *Y* > 0 so that transforms *Y* ^*p*^ are well-defined for all *p*. Model (10) for *Y* ^*p*^ for a fixed power$p$ can be estimated using existing adaptive regression methods as described above. Standard LCV scores for these models, not accounting for the power *p*, as defined earlier are based on the normal density function. The power $p$ can be chosen by maximizing an alternative power-adjusted LCV score that also accounts for power transformation of the outcome *Y* using the power-adjusted likelihood function as defined next.

If *Y* ^*p*^ is normally distributed for *p* > 0, the distribution function *F*(*y*; *p*, ***θ***) for *Y* satisfies

$$ F\left( y; p,\boldsymbol{\theta} \right)= P\left( Y\le y; p,\boldsymbol{\theta} \right)= P\left({Y}^p\le {y}^p;\boldsymbol{\theta} \right) $$

where ***θ*** is the vector of model parameters. Consequently, the power-adjusted density function *f*(*y*; *p*, ***θ***) for *Y* satisfies

$$ f\left( y; p,\boldsymbol{\theta} \right)=\frac{dP\left( Y\le y; p,\boldsymbol{\theta} \right)}{dy}= p\cdot {y}^{p-1}\frac{dP\left({Y}^p\le {y}^p;\boldsymbol{\theta} \right)}{dy} $$

where $\frac{dP\left({Y}^p\le {y}^p;\boldsymbol{\theta} \right)}{dy}$ is the usual univariate normal density function *φ*(*v*, ***θ***) evaluated at *v* = *y* ^*p*^ with mean and variance based on the parameter vector ***θ***. When *p* < 0,

$$ F\left( y; p,\boldsymbol{\theta} \right)= P\left( Y\le y; p,\boldsymbol{\theta} \right)= P\left({Y}^p\ge {y}^p;\boldsymbol{\theta} \right) $$

and the power-adjusted density function *f*(*y*; *p*, ***θ***) for *Y* satisfies

$$ f\left( y; p,\boldsymbol{\theta} \right)=\frac{dP\left( Y\le y; p,\boldsymbol{\theta} \right)}{dy}=- p\cdot {y}^{p-1}\cdot \varphi \left({y}^p;\boldsymbol{\theta} \right). $$

Thus, for *p* ≠ 0,

$$ f\left( y; p,\boldsymbol{\theta} \right)=\left| p\right|\cdot {y}^{p-1}\cdot \varphi \left({y}^p;\boldsymbol{\theta} \right). $$

A similar argument for *p* = 0 corresponding to the natural log transform gives

$$ f\left( y; p,\boldsymbol{\theta} \right)={y}^{-1}\cdot \varphi \left( \log y;\boldsymbol{\theta} \right). $$

The power-adjusted LCV score is defined as

$$ \mathrm{L}\mathrm{C}\mathrm{V}(p)={\displaystyle \prod_{h\in H}}{f}^{\frac{1}{n}}\left( S(h); p,\boldsymbol{\theta} \left( S\backslash S(h); p\right)\right) $$

where *f*(*S*(*h*); *p*, ***θ***(*S*\*S*(*h*); *p*)) denotes the joint power-adjusted likelihood for the subjects with indexes in fold *S*(*h*) computed with estimates ***θ***(*S*\*S*(*h*); *p*) of the parameter vector ***θ*** generated using the data in the complement *S* *S*(*h*) of the fold *S*(*h*) and with the outcome *Y* transformed to *Y* ^*p*^. The LCV(*p*) score can be maximized in the power *p* using a grid search to choose an appropriate power transform for the outcome.

The assumption that *Y* > 0 can be relaxed by extending *Y* ^*p*^ in the same way as for *X* ^*p*^ given earlier, that is, by setting it to 0 when *Y* = 0 and to cos(*π* ⋅ *p*) ⋅ |*Y*|^*p*^ when *Y* < 0. Then, the sign is not always reversed for the case *Y* < 0, affecting the computation of *f*(*y*; *p*, ***θ***). However, the derivative *f*(0; *p*, ***θ***) is not always well-defined. For example, when *p* < 1 and *y* > 0,

$$ f\left( y; p,\boldsymbol{\theta} \right)= p\cdot {y}^{p-1}\cdot \varphi \left({y}^p;\boldsymbol{\theta} \right)\uparrow \infty\ \mathrm{a}\mathrm{s}\ \mathrm{y}\downarrow 0. $$

It seems better to add a constant to *Y* to make it positive valued, which is the approach recommended by Royston and Altman [33] for transforming non-positive predictors.

The univariate outcome transformation process can be applied as well to model (9). The formulation also extends readily to multivariate data. When those data are based on repeatedly measuring the same outcome *Y* at different times or over different conditions (such as members of the same family or patients of the same provider), it is reasonable to transform each such outcome measurement with the same power *p*. However, model (12) requires consideration of different powers *p* and *q* for *Y* and *M* with associated power-adjusted LCV scores LCV(*p*, *q*).

Identification of appropriate choices for $p$ and *q* can be achieved by starting with *p* = *q* = 1 and using grid searches to adjust *p* with *q* = 1 fixed giving LCV(*p*_1_, 1) and to adjust *q* with *p* = 1 fixed giving LCV(1, *q*_1_). If LCV(*p*_1_, 1) > LCV(1, *q*_1_), use a grid search in *q* with *p* = *p*_1_; otherwise use a grid search in *p* with *q* = *q*_1_. This generates powers *p*_2_ and *q*_2_. In example analyses, the grid searches are first conducted over changes of ±0.5 generating the powers *p*_2_ and *q*_2_, then over changes of ±0.1 starting at the powers *p*_2_ and *q*_2_ generating the powers *p*_3_ and *q*_3_, and then stops identifying powers for *Y* and *M* to within one decimal digit.

#### Monotonic mediation analysis

In what follows, unless otherwise stated, base models for means and covariances are based on the predictors ***I***_1_ and ***I***_2_, and so with only intercepts *i*_*M*_ and *i*_*Y*_ for the means along with CSH covariance structure. Also assume *p* " = 1 unless otherwise indicated.

1. Selecting the number *k* of folds.
  The benchmark analysis is the generation of model (12) constrained so that *p* = *q* = 1, that is, with untransformed *M* and *Y*, by adaptively expanding the model for the means in ***X***_1_ and ***X***_2_ limiting the number of power transforms for each of these predictors to one. The number of folds to use in all subsequent analyses is the one generating the first local maximum in the standard LCV score over multiples of 5.
2. Selecting the powers *p* and *q*.
  Use the search through alternative values for *p* and *q* based on power-adjusted LCV scores LCV(*p,q*) described earlier to generate the full model (12) without changing the CSH covariance structure. Use the selected powers *p* and *q* in subsequent analyses unless otherwise indicated.
3. Assessing the need for transforming *M* and *Y*.
  Use a LCV ratio test to compare LCV(1,1) (the same as its standard LCV score) generated in Step 1 to LCV(*p*,*q*) generated in Step 2.
4. Assessing the need for transforming *X*.
  For given values of *p* and *q*, use a LCV ratio test to compare LCV(*p*,*q*) as generated in Step 2, with ***X***_1_ and ***X***_2_ adaptively transformed, to the LCV(*p*,*q*) score for the associated model linear in ***X***_1_ and ***X***_2_.
  When the power *q* ' = 1, the instantaneous natural indirect effect $\frac{dNIE(x)}{dx}= a\cdot b$ is constant. Whether the instantaneous natural indirect effect is reasonably treated as constant can be assessed with a LCV ratio test comparing a given model with *q* ' ≠ 1 to that model adjusted to satisfy *q* ' = 1.
5. Assessing mediation relationships.
  In the linear mediation case of models (1)-(3), mediation requires significantly nonzero slopes *a* and *b*. In the monotonic mediation context of model (12), these issues can be addressed with LCV ratio tests. To assess for a dependence of *M* ^*q*^ on *X* in the component model (9), compare the LCV(*p*,*q*) score for the full model (12) to the associated model with ***X***_1_ removed (or with *a* = 0) and the transform for ***X***_2_ adaptively generated. To assess the dependence of *Y* ^*p*^ on *M* ^*q*^ in the component model (10) with *p* " = 1, compare the LCV(*p*,*q*) score for the full model (12) to the associated model with ***M*** ^*q*^ removed (or with *b* = 0) and the transforms for ***X***_1_ and ***X***_2_ adaptively generated. It is also possible to assess the dependence of *Y* ^*q*^ on $X$ in the component model (10) by comparing the LCV(*p*,*q*) score for the full model (12) to the associated model with ***X***_2_ removed (or with *c* ' = 0) and the transform for ***X***_1_ adaptively generated.
6. Considering non-constant variances.
  With base model the adaptive model (12) with CSH covariance structure generated in Step 2 with initial powers *p* ' and *q* ' for ***X***_1_ and ***X***_2_, adaptively expand and then contract the model for the variances in ***X***_1_, ***X***_2_, and ***M***, allowing for possible adjustment of the powers *p* ' and *q* ' of the base model as part of the contraction, but leaving ***M*** ^*q*^ in the base model untransformed (so that *p* " = 1).
7. Considering covariates.
  Covariates can be considered for inclusion in the component models (9)-(10) of model (12). With base model the adaptive model of Step 2, adaptively expand and then contract the model for the means and the variances in ***Z***_1_ and ***Z***_2_ for all covariates *Z*. As part of the contraction, allow for possible adjustment of the powers *p* ' and *q* ' of the base model, leave ***M*** ^*q*^ in the base model untransformed (so that *p* " = 1), and only contract transforms of covariates from the model for the means. This can be combined with Step 6 to allow the variances to depend as well on ***X***_1_, ***X***_2_, and ***M***.
8. Assessing *ρ* = 0.
  For any model generated earlier, compare its LCV score using a LCV ratio test to the associated model constrained to satisfy *ρ* = 0.
9. Assessing *p* " = 1
  With base model the adaptive model of Step 2, adaptively retransform the model allowing the powers of the single transforms of ***X***_1_, ***X***_2_, and ***M*** ^q^ to be changed to improve the LCV score. The assumption *p* " = 1 is supported if the associated model generated with *p* " = 1 in Step 2 is a competitive alternative to this latter model. Similar assessments can be conducted allowing for non-constant variances as in Step 6, for covariates as in Step 7, and/or *ρ* = 0 as in Step 8.
10. Assessment of Model Assumptions
  Yuan and MacKinnon [26] provide a detailed discussion of the impact of violations of the constant variances and normality assumptions of standard regression models. Modeling of variances can address the first problem. Data transformation, applied to outcomes and/or predictors, as considered here can sometimes resolve normality problems, but not always. Consequently, model assumption assessments are important to conduct in general regression contexts including the special case of mediation analyses, whether treated as linear or nonlinear. Should data transformation not resolve such problems, then quantile regression methods as described by Yuan and MacKinnon [26] are more appropriate to use. However, if data transformation resolves model assumption problems, then normality-based methods are optimal [26] when applied to the transformed data and so would likely generate more efficient and powerful estimates.
  In the case of mediation analyses, the use of bootstrapped CIs on indirect effects circumvents distributional assumption problems for parametric estimates of those effects. However, as demonstrated by the simulations of [26], bootstrap methods cannot fully address distributional assumption problems for the data. Moreover, bootstrapped CIs are likely to be relatively narrower, and so more precise, when data are transformed to be as close as possible to normal, than when untransformed. The example analyses reported later provide two examples supporting this conclusion. This, of course, assumes that the indirect effect has been consistently estimated so that the true value is in the confidence interval.
  For composite model (12), the constant variances assumption can be addressed with a LCV ratio test comparing constant and non-constant variances models to assess whether variances are reasonably close to constant or are distinctly non-constant. If a sufficiently broad set of primary predictors for the variances are considered, the associated non-constant variances model should provide an appropriate depiction of the variances, thereby relaxing the constant variances assumption if necessary. These variances estimates combined with the estimated correlation provide estimates **Σ**_*s*_(*S*) of the covariance matrices for observed outcome vectors ${\boldsymbol{Y}}_{\boldsymbol{s}}^{\mathit{\hbox{'}}}$ for subjects with indexes *s* in the set *S*. Associated residual vectors are ***u***_***s***_(***S***) = ${\boldsymbol{Y}}_{\boldsymbol{s}}^{\mathit{\hbox{'}}}$ − ***μ***_***s***_(***S***) where ***μ***_***s***_(***S***) are estimated mean vectors for subjects $s$ based on ***X***_1*s*_, ***X***_2*s*_, ***M***_s_, and possibly covariates. Associated standardized or scaled residuals are given by ***stdu***_*s*_(*S*) = (***V***_*s*_^*T*^(*S*))^− 1^ · ***u***_*s*_(*S*) where ***V***_*s*_(*S*) is the square root of **Σ**_*s*_(*S*) determined by its Cholesky decomposition. The combined standardized residuals over all *s* can be used to assess the normality assumption by visually checking for linearity in the associated normal (probability) plot and with the Shapiro-Wilk test for normality of the standardized residuals.
11. Assessing natural indirect effects
  Once an appropriate choice for composite model (12) with *p* " = 1 has been identified, possibly including non-constant variances and/or covariates, the assessment of whether the instantaneous natural indirect effect function $\frac{dNIE(x)}{dx}$ for this model is nonzero needs to be assessed. This can be addressed with bootstrapped CIs [21, 29, 40, 41], but computed for a grid of possible values *x* for the predictor *X*. For models with a constant instantaneous natural indirect effect (i.e., with *q* ' = 1), only one value for *X* need be considered. The bias-corrected version [42] is recommended by MacKinnon et al. [40], and so is used in example analyses unless otherwise indicated. All reported CIs are based on 1,000 resamples.
  The powers *p*, *q*, *p* ', and *q* ' for a composite model (12) as well as powers for all covariate predictors and variance predictors are held fixed with associated slope parameters estimated for resamples of the composite data. The generated 95% CI for the instantaneous natural indirect effect function at each nonzero value of $x$ has lower and upper bounds
  $$ L(x)={b}_L\cdot {a}_L\cdot q\mathit{\hbox{'}}\cdot {x}^{q\mathit{\hbox{'}}-1}< U(x)={b}_U\cdot {a}_U\cdot q\mathit{\hbox{'}}\cdot {x}^{q\mathit{\hbox{'}}-1}. $$
  Define the normalized width *W* of these intervals as
  $$ W=\frac{ \max \left(\left| L(x)\right|,\left| U(x)\right|\right)- \min \left(\left| L(x)\right|,\left| U(x)\right|\right)}{ \max \left(\left| L(x)\right|,\left| U(x)\right|\right)}=1-\frac{ \min \left(\left|{b}_L\cdot {a}_L\right|,\left|{b}_U\cdot {a}_U\right|\right)}{ \max \left(\left|{b}_L\cdot {a}_L\right|,\left|{b}_U\cdot {a}_U\right|\right)}, $$
  which is constant in nonzero $x$ with a value between 0 and 1. Models for the data generating smaller values for *W* provide more precise predictions of the instantaneous natural indirect effect function.

#### Moderated monotonic mediation

One of the covariates *Z* might be considered as a moderator. There are a variety of ways that models (9)-(10) can be adjusted to accommodate moderation. For example, Preacher, Rucker, and Hayes [53] propose five alternatives. Under their fifth alternative, *Z* moderates the effect of *X* on *M*, *X* on *Y*, and *M* on *Y*. Under this alternative, models (9)-(10) become  possibly with other covariates included where  is the set of all possible values *z* for *Z* and *I*(*Z* = *z*) the indicator for *Z* = *z* (i.e., it equals 1 when *Z* = *z* and 0 otherwise). The dependence of *M* on *X* and *Y* on *X* and *M* have been defined separately for the values *z* of *Z* taking an analysis of variance approach. This formulation allows associated intercepts, slopes, and powers to change with the values *z* of *Z* while preserving monotonicity. However, it requires estimation of model parameters for each value *z* of Z. This requirement is reasonable for moderators *Z* with discrete numbers of possible values, but problematic for continuous moderators *Z* with many possible values *z* but sparse numbers of observations for some values *z*. In this latter case, the moderator *Z* could be replaced by a split based on its tertiles, quartiles, etc.

Under (13)-(14), the instantaneous natural direct effect ${c}^{\mathit{\hbox{'}}}( z)\cdot {p}^{\mathit{\hbox{'}}}( z)\cdot {X}^{p^{\mathit{\hbox{'}}}(z)-1}$ (assuming for simplicity that *p* ' (*z*) ≠ 0) changes with the values *z* of *Z*. When *p* " (*z*)≡1, the instantaneous natural indirect effect *b*(*z*) ⋅ *a*(*z*) ⋅ *q*′(*z*) ⋅ *X* ^(*q* ' (*z*) − 1)^ (assuming for simplicity that *q*′(*z*) ≠ 0) also changes with the values *z* of *Z*. The associated normalized widths W(*z*) change with *z* as well.

Model (12) generalizes to  where

$$ \boldsymbol{H}( z)={\beta}_1( z)\cdot {\boldsymbol{I}}_1+{\beta}_2( z)\cdot {\boldsymbol{I}}_2+{\beta}_3( z)\cdot {\boldsymbol{X}}_1^{q^{\prime }(z)}+{\beta}_4( z)\cdot {\boldsymbol{X}}_2^{p^{\prime }(z)}+{\beta}_5( z)\cdot {\boldsymbol{M}}^q. $$

The adaptive modeling process can be used to identify the powers *q* ' (*z*) and *p* ' (*z*) for all *z* combined.

The individual moderation components of model (15) can be assessed by comparing model (15) to the model with each of those moderation component removed using LCV ratio tests. Specifically, moderation of the effect of *X* on *M* in model (13) can be assessed by replacing  in (15) with *β*_3_ ⋅ ***X***_1_^*q* '^. Moderation of the effect of *X* on *Y* in model (14) can be assessed by replacing  in (15) with *β*_4_ ⋅ ***X***_2_^*p* '^. Moderation of the effect of *M* on *Y* in model (14) can be assessed by replacing  in (15) with *β*_5_ ⋅ ***M*** ^*q*^.

It is also possible to test effects for specific values *z* ' of *Z*. Specifically, the effect of *X* on *M* in model (13) for the value *z*′ of *Z* can be assessed by replacing  in (15) with . The effect of *X* on *Y* in model (14) for the value$z^{\prime }$ of *Z* can be assessed by replacing  in (15) with . The effect of *M* on *Y* in model (14) for the value *z*′ of *Z* can be assessed by replacing  in (15) with .

### Data on family management of childhood chronic conditions

Example analyses are reported later using a subset of data from a cross-sectional study on family management of childhood chronic conditions [54] reported by 187 partnered mothers. General family functioning is measured using the General Functioning Scale of the McMaster Family Assessment Device [55], coded so that larger values indicate better family functioning with range 1–4. Difficulty managing the child’s condition is measured by the family life difficulty scale of the Family Management Measure [54], coded so that larger scores mean more difficulty. This scale measures the extent to which having a child with a chronic condition makes family life more difficult. Child adaptation, in terms of the intensity of the child’s conduct-disordered behavior, is measured using the Eyberg Child Behavior Inventory [56], coded so that larger values indicate better child adaptation or less conduct-disordered behavior.

Example analyses use these family management data to demonstrate nonlinear mediation analyses by considering mediation of the impact of family functioning *X* on child adaptation *Y* by difficulty *M* in managing the condition. The cutoff for a substantial percent decrease for these data using models (12) and (15) with 374 = 2 ⋅ 187 measurements is 0.51%. Example analyses assume *p* " = 1 or *p* " (*z*) = 1 for all values *z* of a moderator *Z* unless otherwise stated.

The proposed mediation relationships for these observational data can be justified on the following basis. General family functioning would be developed by a family prior to the diagnosis of the child’s chronic condition, which would affect how difficult that chronic condition makes family life which would then affect the child’s adaption to the condition. However, the purpose of these analyses is to provide example mediation analyses not to establish mediation in this context.

### Simulated mediation data

Data were simulated for 101 observations with equally spaced values for the predictor *X*_*sim*_ between 1 and 10 (i.e., 0.09 units apart) with mediator *M*_*sim*_ = 1 + *X*_*sim*_ + *U*_*M*_, outcome *Y*_*sim*_ = *Y* ' _*sim*_^0.4^, and

$$ Y{\mathit{\hbox{'}}}_{sim}=\frac{5+{X}_{sim}+{M}_{sim}+{U}_Y}{25}, $$

where *U*_*M*_ and *U*_*Y*_ are independent standard normal random variables. *Y*_*sim*_ was computed by raising *Y* ' _*sim*_ to the power 2.5; the normalizing value 25 used in computing *Y* ' _*sim*_ was chosen to be the smallest integer value larger than the maximum generated value for the unnormalized *Y* ' _*sim*_ values. Normalizing the values of *Y* ' _*sim*_ avoids generating very large values for *Y*_*sim*_. The true values for the powers are *p* = 0.4, *q* = 1, *p* ' = 1, and *q* ' = 1 with true constant instantaneous natural indirect effect $$ a\cdot b=1\cdot \raisebox{1ex}{$1$}\!\left/ \!\raisebox{-1ex}{$25$}\right.=0.04 $$. The cutoff for a substantial percent decrease for these data using model (12) with 202 = 2 ⋅ 101 measurements is 0.95%.

## Results

Analyses at the beginning of this section consider mediation of the effect of family functioning on child adaptation to a childhood chronic condition by the difficulty in managing that condition. Intercept parameters for the means and variances are constrained in all analyses not to be removed as part of contractions. In computing LCV scores, *Y* ^*p*^ and *M* ^*q*^ measurements for the same mother are randomly assigned to the same fold. Analyses are also conducted at the end of this section using the simulated mediation data.

### Selecting the number of folds

For model (12) applied to the family management data with *p* = *q* = 1 and CSH covariance structure, the adaptive model in ***X***_1_ and ***X***_2_ generates a first local maximum at *k* = 10 with $10$-fold LCV score $0.015024$ and selected powers *p* ' = −3 and *q* ' = 2. Using *k* = 5, the selected powers are *p* ' = 3 and *q* ' = 1.5 with $10$-fold LCV $0.015018$ and insubstantial percent decrease in the LCV score compared to the model selected with *k* = 10 of 0.04% (i.e., less than the cutoff of 0.51% for the data). Using *k* = 15, the selected powers are *p* ' =−1 and *q* ' = 1.5 with $10$-fold LCV $0.015005$ and insubstantial percent decrease in the LCV score of 0.13%. Consequently, the generated model is reasonably robust to the choice of the number of folds. Subsequent analyses all use *k* = 10 folds for computing standard as well as power-adjusted LCV scores.

Using$10$ folds, the number of measurements per fold ranges from $26$ to $56$ for $13$ to $28$ mothers. Consequently, fold complements contain at least $318$ or $85.0\%$ of the $374$ available measurements, and so deleted parameter estimates should be reasonably reliable.

### Selecting the powers $p$ and $q$ for $Y$ and $M$

For model (12) with CSH covariance structure, the adaptive search for models in ${\boldsymbol{X}}_1$ and ${\boldsymbol{X}}_2$ with varying choices for $p$ and $q$, first selects the powers *p* = 1 and *q* = 0 (i.e., the log transform) over changes in these powers of ±0.5 with LCV(1, 0) = 0.015683 and then the powers *p* = 1.3 and *q* = 0 over changes in these powers of ±0.1 with LCV(1.3,0) = 0.015776. For untransformed *Y* and *M* with *p* = *q* = 1, LCV(1, 1) = 0.015024 (the same as its standard LCV score reported above), and so the percent decrease is substantial at $4.77\%$. Consequently, the mediation relationships are distinctly nonlinear in $Y$ and $M$. Model (12) for the standard linear mediation model with *p* = *q* = *p* ' = *q* ' = 1 has even smaller LCV(1,1) score 0.014958, and so is substantially improved upon by consideration of monotonicity.

Furthermore, the adaptively generated model with$Y$ transformed and $M$ held untransformed, that is, with *q* = 1, generates the powers *p* = 1.4, *p* ' = −5, and *q* ' = 2 with LCV(1.4,1) score 0.015143 and substantial percent decrease compared to the model with *p* = 1.3 and *q* = 0 of 4.01%. Consequently, relationships (9)-(10) are distinctly nonlinear in $M$. Moreover, the adaptively generated model with $Y$ untransformed, that is, with *p* = 1, generates the powers *q* = 0, *p* ' = −2, and *q* ' = 2.4 with LCV(1,0) score 0.15683 and substantial percent decrease compared to the model with *p* = 1.3 and *q* = 0 of 0.59%. Hence, relationship (10) is distinctly nonlinear in *Y*.

### Assessing ***ρ*** = 0

Using *p* = 1.3, *q* = 0, *p* ' = −2.5, and *q* ' = 3.5 as selected with the CSH covariance structure, the estimated correlation is −0.08. Rerunning this model with ***ρ*** = 0, the LCV(1.3,0) score is 0.015804. Using *p* = 1.3 and *q* = 0 as selected with CSH covariance structure, but with *ρ* = 0, the adaptively generated model (12) has somewhat different powers *p* ' = −3 and *q* ' = 3 and LCV(1.3,0) score that also rounds to 0.015804. Since this score is larger than the LCV(1.3,0) score under CSH, the omitted variables or errors *U*_*M*_ and *U*_*Y*_ are reasonably treated as independent. Using the values of *p* and *q* selected under CSH reduces the computations compared to identifying adaptive values for *p* and *q* under *ρ* = 0. However, in this case, the same powers *p* = 1.3 and *q* = 0 are adaptively identified with *ρ* = 0 starting from *p* = *q* = 1. An alternative approach would be to start the search for *p* and *q* with *ρ* = 0 at the values generated for CSH while searching over grids of $\pm 0.1$ to reduce the computations. This also generates the same solution $p=1.3$ and $q=0$.

For the case $p= q=1$ with selected powers ${p}^{\hbox{'}}=-3$ and ${q}^{\hbox{'}}=2$, a similar result holds. The estimated correlation is $-0.05$ and the model with $\rho =0$ has LCV score $0.015043$, larger than the score $0.015024$ reported earlier for the associated model with CSH covariance structure. Subsequent analyses use $\rho =0$ since it provides an improvement in these two cases.

### Assessing ***a*** = 0

The adaptively generated model with $p=1.3$, $q=0$, $\rho =0$, and constrained not to include a transform of ${\boldsymbol{X}}_1$ in the model for the means has model for the means depending on ${\boldsymbol{X}}_2$ transformed by the power *p*' = −3 with LCV(1.3,0) score $0.015077$ and substantial percent decrease $4.60\%$ compared to the model also including a transform of ${\boldsymbol{X}}_1.$ Consequently, transformed difficulty $\log M$ in model (9) is reasonably assumed to depend substantially on family functioning $X$.

### Assessing $b=0$

The adaptively generated model with $p=1.3$, $q=0$, $\rho =0$, and constrained not to include the transform ${\boldsymbol{M}}^q$ in the model for the means has model for the means depending on ${\boldsymbol{X}}_1$ and ${\boldsymbol{X}}_2$ transformed by the powers *p*′ = −0.4 and *q*′ = 3, respectively, with LCV(1.3,0) and substantial percent decrease $1.07\%$ compared to the model also including the transform of ***M*** ^*q*^. Consequently, transformed adaptation ${Y}^{1.3}$ in model (10) is reasonably assumed to depend substantially on transformed difficulty $\log M$ corresponding to $q=0$.

### Assessing ***c*** ' = 0

The adaptively generated model with $p=1.3$, $q=0$, $\rho =0$, and constrained not to include a transform of ${\boldsymbol{X}}_2$ in the model for the means has model for the means depending on ${\boldsymbol{X}}_1$ transformed by the power *q*' = 3 with LCV(1.3,0) and substantial percent decrease $0.57\%$ compared to the model also including a transform of ${\boldsymbol{X}}_2$. Consequently, in model (10), transformed adaptation ${Y}^{1.3}$ is reasonably assumed to depend substantially on family functioning $X$, and so the instantaneous natural direct effect is substantial.

### Assessing *p* " = 1

Using the model (12) selected with $p=1.3$, $q=0$, and $\rho =0$ (i.e., with *p*' = −3 and *q*' = 3), an adaptive transformation of this model allowing for *p*" ≠ 1 (and also possible adjustments to *p*' and *q*') is the same model generated with *p*" = 1. Consequently, the simplifying assumption *p*" = 1 is reasonable in this case.

### Assessing non-constant variances

As described earlier, adaptive models can be generated allowing for variances of the omitted factors or errors ${U}_M$ and ${U}_Y$ to depend on transforms of ${\boldsymbol{X}}_1$, ${\boldsymbol{X}}_2$, and $\boldsymbol{M}$. Starting from the adaptively generated model for $p=1.3$ and $q=0$ with $\rho =0$ (i.e., with *p*' = −3 and *q*' = 3), the expansion adds in transforms of these predictors to the model for the variances, but the contraction removes all of them, leaving the base constant variances model. This result indicates that the variances are reasonably treated as constant in ${\boldsymbol{X}}_1$, ${\boldsymbol{X}}_2$, and $\boldsymbol{M}$.

### Also considering a covariate

The family management study enrolled parents of children with a variety of chronic conditions. One childhood chronic condition type was Crohn’s disease or a bowel disorder with $54$ ($28.9\%$) children having this condition. The indicator $Z$ for having this condition can be considered as a possible covariate for models (9)-(10). Adaptive modeling starts with the adaptively generated model for $p=1.3$, $q=0$, and $\rho =0$ (i.e., with *p*' = −3 and *q*' = 3) along with constant variances (and so based on ${\boldsymbol{I}}_1$ and ${\boldsymbol{I}}_2$). The model for the means is expanded considering the indicators ${\boldsymbol{Z}}_1$ and ${\boldsymbol{Z}}_2$ (as defined similarly to ${\boldsymbol{X}}_1$ and ${\boldsymbol{X}}_2$) while the model for the variances is expanded considering arbitrary transforms of ${\boldsymbol{X}}_1$, ${\boldsymbol{X}}_2$, and $\boldsymbol{M}$ along with ${\boldsymbol{Z}}_1$ and ${\boldsymbol{Z}}_2$. The contraction is constrained so that ${\boldsymbol{M}}^q$ is not retransformed in the model for the means (so ${p}^{\hbox{'}\hbox{'}}=1$) while ${\boldsymbol{X}}_1$ and ${\boldsymbol{X}}_2$ are not removed from the model for the means, but associated powers are allowed to be changed.

The generated model has the same powers *p*' = −3 and *q*' = 3 as without consideration of ${\boldsymbol{Z}}_1$ and ${\boldsymbol{Z}}_2$ along with the covariate ${\boldsymbol{Z}}_2$ added to both models for the means and the variances and no transforms of ${\boldsymbol{X}}_1$, ${\boldsymbol{X}}_2$, and $\boldsymbol{M}$ in the model for the variances. The LCV(1.3,0) score is $0.016062$, which is a substantial improvement on the score $0.015804$ for the associated model not considering covariates with percent decrease $1.61\%$. Consequently, the indicator for having Crohn’s disease or a bowel disorder substantially influences the means and variances for model (10), but not for model (9) (since only ${\boldsymbol{Z}}_2$ is included in the generated model and not ${\boldsymbol{Z}}_1$).

### Model assumptions

**Fig. 3:**
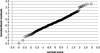
Normal plot for the linear mediation model for child adaptation as a function of family functioning as mediated by difficulty with independent omitted factors or errors

A standard linear mediation analysis, that is, with model (12) based on *p* = *q* = *p*' = *q*' = 1 and $\rho =0$, generates the standardized residuals plotted in Fig. 3. While this plot is reasonably close to linear for most of the data, there are exceptions at the low and high ends of the plot. There are also three outliers (i.e., with values outside of $\pm 3\Big)$ with standardized residual values of $-3.44$, $3.03$, and $3.07$. The Shapiro-Wilk test for normality of the standardized residuals is significant at $p=0.037$. Consequently, the normality assumption is questionable for the linear mediation model. The corresponding adaptive non-constant variances model with no changes to the model for the means but possible inclusion of transforms of ${\boldsymbol{X}}_1$, ${\boldsymbol{X}}_2$, and $\boldsymbol{M}$ in the model for the variances contains the single transform ${\boldsymbol{X}}_1^{19}$ in the model for the variances. The LCV(1,1) score for this adjusted model is $0.015046$ with substantial improvement over the score $0.014958$ for the associated constant variances model (as reported earlier) with percent decrease $0.58\%$. Consequently, the constant variance assumption is questionable for the component model (2) of the standard linear moderation model.

**Fig. 4:**
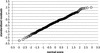
Normal plot for the monotonic mediation model for child adaptation as a function of family functioning as mediated by difficulty controlling for having Crohn’s disease or a bowel disorder with independent omitted factors or errors

**Fig. 5:**
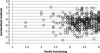
Standardized residual plot for the monotonic mediation model for child adaptation as a function of family functioning as mediated by difficulty controlling for having Crohn’s disease or a bowel disorder with independent omitted factors or errors

Figure 4 contains the normal plot generated by the monotonic mediation model (with $p=1.3$, $q=0$, *p*' = −3, *q*' = 3, and $\rho =0$) adjusted for the covariate having Crohn’s disease or a bowel disorder with the best power-adjusted LCV score generated so far. This plot is reasonably close to linear, the standardized residuals range for $-3.01$ to $2.51$ with only one observation having a value $-3.01$ outside of $\pm 3$, but very close to the boundary value of $-3$. The Shapiro-Wilk test for normality is now non-significant ($p=0.475$), and so the normality assumption seems reasonable for this case. Figure 5 contains the plot of the standardized residuals in terms of family functioning. The assumption of constant variances is reasonable at least for all but a few exceptional, low family functioning values, suggesting that the standardized residuals are reasonable close to having constant variances. These results indicate that monotonic transformation can resolve distributional assumption problems for mediation models with continuous positive valued outcomes and mediators.

### Results for the selected model

Using this monotonic mediation model with the best LCV score so far, estimated instantaneous total, natural direct, natural indirect, and relative natural indirect effects are presented in Table 1 for the grid of family functioning values 1, 2, 3, and 4 (1 is the smallest possible value while 4 is the largest possible value for the scale). The instantaneous natural indirect effect of family functioning on child adaptation increases with increasing or improving family functioning values while the instantaneous natural direct effect of family functioning on child adaptation decreases in such a way that the relative instantaneous natural indirect effect of family functioning on child adaptation increases. Family functioning needs to be relatively high in order for the relative instantaneous natural indirect effect to be relatively strong; in other words, mediation in this case is quite weak for values of family functioning between 1 and 2 In contrast, the standard linear mediation model (i.e., with *p* = *q* = *p*' = *q*' = 1 and $\rho =0\Big)$ generates a total effect of 18.5, a natural direct effect of 12.3, a natural indirect effect of 6.2, and a relative natural indirect effect of 0.34, quite different results.

**Table 1:** Estimated instantaneous total, natural direct, natural indirect, and relative natural indirect effects for the monotonic mediation model for child adaptation as a function of family functioning as mediated by difficulty controlling for having Crohn’s disease or a bowel disorder with independent omitted factors or errors

Family functioning	Instantaneous total effect	Instantaneous natural direct effect	Instantaneous natural indirect effect	Relative instantaneous natural indirect effect
1	3092.9	3087.9	4.9	0.002
2	212.6	193.0	19.6	0.092
3	82.3	38.1	44.1	0.536
4	90.5	12.1	78.5	0.867

### Bootstrapped CIs

The standard linear mediation model (i.e., with *p* = *q* = *p*' = *q*' = 1 and $\rho =0$) generates the same bootstrapped CI of $2.00$ – $11.96$ for the instantaneous natural indirect effect at each value of family functioning. The normalized width $W$ for this CI is $0.83$.

Table 2 contains bias-corrected bootstrapped $95\%$ CIs for the natural indirect effect at a range of values of family functioning under the monotonic mediation model with the best LCV score so far, that is, model (12) with powers $p=1.3$, $q=0$, *p*' = −3, and *q*' = 3, controlling for the covariate having Crohn’s disease or a bowel disorder with independent omitted factors or errors (i.e., $\rho =0$). The lower and upper bounds on the instantaneous natural indirect effects of family functioning on child adaptation increase with increasing values of family functioning. The normalized width $W$ for these CIs is $0.72$. Since this is smaller than the value $0.83$ (about $13\%$ smaller) for the standard linear mediation model, the monotonic mediation model generates more precise estimates of the instantaneous natural indirect effects.

**Table 2:** Bias-corrected bootstrapped $95\%$ confidence intervals for the monotonic mediation model for child adaptation as a function of family functioning as mediated by difficulty controlling for having Crohn’s disease or a bowel disorder with independent omitted factors or errors

Family functioning x	Lower bound $L(x)$ on natural indirect effect^a^	Upper bound $U(x)$ on natural indirect effect^a^	Normalized width $W$ of the confidence interval^b^
1	2.28	8.19	0.72
2	9.14	32.76	0.72
3	20.56	73.72	0.72
4	36.55	130.05	0.72

^a^Using 1,000 resamples

^b^ *W* = (*U*(*x*) − *L*(*x*))/*U*(*x*), which is constant in $x$

The bootstrapped CI of $2.00$ – $11.96$ for the linear moderation model (as reported earlier) overlaps with the bootstrapped CIs for $x=1$ and $x=2$, but is entirely below the bootstrapped CIs for $x=3$ and $x=4$, suggesting that the linear moderation model in this case generates biased estimates of natural indirect effects for larger values of family functioning.

### Moderated monotonic mediation

Another childhood chronic condition type for the family management study was diabetes with $51$ ($27.3\%$) children having this condition. The indicator $Z$ for having this condition can be considered as a possible moderator for models (13)-(15). As before, the indicator for the child having Crohn’s disease or a bowel disorder is considered as a covariate to include in both the models for the means and the variances as well as the model for the variances to depend on transforms of ${\boldsymbol{X}}_1$, ${\boldsymbol{X}}_2$, and $\boldsymbol{M}$.

The generated model for the case of children with a chronic condition other than diabetes ($Z=0$) has powers *p*'(0) = −1.7 and *q*'(0) = 6. The generated model for the case of children with diabetes ($Z=1$) has the power *q*′(1) = 0.5 with no transform of family functioning (so a missing *p*′(1) power). As before the covariate Crohn’s disease or a bowel disorder is included in the model for the means and variances, but only in the component of model (15) addressing the outcome variable, that is, submodel (14). The LCV(1.3,0) score is $0.016215$, which is a substantial improvement on the score $0.016062$ for the associated model not considering moderation with percent decrease $0.94\%.$ Consequently there is distinct moderated mediation.

The linear moderated mediation model (i.e., with *p* = *q* = 1, *p*'(0) = *q*(0) = 1, *p*'(1) = *q*(1) = 1, and $\rho =0$) allowing for effects of the covariate having Crohn’s disease or a bowel disorder on the means and variances as well as possibly nonlinear effects of ${\boldsymbol{X}}_1$, ${\boldsymbol{X}}_2$, and $\boldsymbol{M}$ on the variances has means and variances depending on the covariate having Crohn’s disease of a bowel disorder for only the submodel (14) as for the moderated monotonic mediation model. Its LCV(1,1) score is $0.015460$ with substantial percent decrease $4.66\%$. Consequently, the moderated mediation is distinctly nonlinear.

Model (15) adjusted to remove moderation of the effect of transformed $X$ on transformed $M$ in submodel (13) has LCV(1.3,0) score $0.016112$ with substantial percent decrease $0.64\%$ compared to the full model (15). Moreover, model (15) adjusted to remove moderation of the effect of transformed $X$ on transformed $Y$ in submodel (14) has LCV(1.3,0) score $0.016112$ (same score as above but a different model) with substantial percent decrease $0.64\%$ compared to the full model (15). Consequently, there is distinct moderation of both effects of the predictor $X$ in model (15).

On the other hand, model (15) adjusted to remove moderation of the effect of transformed M on transformed Y in submodel (14) has LCV(1.3,0) score $0.016155$ with insubstantial percent decrease $0.37\%$ compared to the full model (15). Consequently, this is a parsimonious, competitive alternative model, and the effect of transformed $M$ on transformed $Y$ is reasonably considered not to be moderated by having diabetes.

Model (15) with the term *β*_3_(0) ⋅ *X*_1_^*q* ' (0)^ ⋅ *I*(*Z* = 0) removed has powers *q* ' (1) = 0.5 and *p* ' (0) = −1 with LCV(1.3,0) score $0.015846$ and substantial percent decrease $2.28\%$. Thus, there is a distinct effect of transformed $X$ on transformed $M$ for children with a chronic condition other than diabetes in submodel (13). With the term *β*_5_(0) ⋅ ***M*** ^*q*^ ⋅ *I*(*Z* = 0) removed, the model has powers *q*'(0) = 6, *q* ' (1) = 0.5, and *p* ' (0) = 0.5 with LCV(1.3,0) score $0.015949$ and substantial percent decrease $1.64\%$. Thus, there is a distinct effect of transformed $M$ on transformed $Y$ for children with a chronic condition other than diabetes in submodel (14).

Model (15) with the term *β*_3_(1) ⋅ ***X***_1_^*q* ' (1)^ ⋅ *I*(*Z* = 1) removed has powers *q*'(0) = 6 and *p*'(0) = −1 with LCV(1.3,0) score $0.015674$ and substantial percent decrease $3.34\%$. Thus, there is a distinct effect of transformed $X$ on transformed $M$ for children with diabetes in submodel (13). On the other hand, with the term *β*_5_(1) ⋅ ***M*** ^*q*^ ⋅ *I*(*Z* = 1) removed, the model has powers *q*'(0) = 6, *q*'(1) = 0.5, and *p*'(0) = −1 with LCV(1.3,0) score $0.016137$ and insubstantial percent decrease $0.48\%$. Thus, the effect of transformed $M$ on transformed $Y$ for children with diabetes in submodel (14) is not distinct. This latter result indicates that the effect of transformed $X$ on transformed $Y$ is not distinctly mediated by transformed $M$ for children with diabetes.

With the term ${\beta}_4(0)\cdot {\boldsymbol{X}}_2^{p^{\hbox{'}}(0)}\cdot I\left( Z=0\right)$ removed, the model has powers *q*'(0) = 6 and *q*'(1) = 0.5 with LCV(1.3,0) score $0.016058$ and substantial percent decrease $0.97\%$. This result indicates that there is a distinct effect of transformed $X$ on transformed $Y$ for children with a chronic condition other than diabetes in submodel (14). On the other hand, the fact that ${\beta}_4(1)\cdot {\boldsymbol{X}}_2^{p^{\hbox{'}}(1)}\cdot I\left( Z=0\right)$ is not in the generated model indicates that the effect of transformed $X$ on transformed $Y$ for children with diabetes in submodel (14) is not distinct.

Using the parsimonious, competitive model with the term *β*_5_(1) ⋅ ***M*** ^*q*^ ⋅ *I*(*Z* = 1) removed, the standardized residuals range from $$ -2.89 $$ to $$ 2.48 $$ with nonsignificant ($$ p=0.430 $$) Shapiro-Wilk normality test and normal plot reasonably close to linear (not displayed). Table 3 contains the associated estimated instantaneous total, natural direct, natural indirect, and relative natural indirect effects for the grid of family functioning values 1, 2, 3, and 4, but just for families having children with chronic conditions other than diabetes. The instantaneous natural indirect effect of family functioning on child adaptation increases with increasing or improving family functioning values while the instantaneous natural direct effect of family functioning on child adaptation decreases in such a way that the relative instantaneous natural indirect effect of family functioning on child adaptation increases. Compared to Table 1, instantaneous natural indirect effects and instantaneous total effects are smaller for low values of family functioning (1–$$ 3) $$ and larger than for the highest value of family functioning (4). Relative instantaneous natural indirect effects are all smaller.

**Table 3:** Estimated instantaneous total, natural direct, natural indirect, and relative natural indirect effects for the monotonic mediation model for child adaptation as a function of family functioning as mediated by difficulty controlling for having Crohn’s disease or a bowel disorder and as moderated by having diabetes with independent omitted factors or errors

Diabetes^a^	Family functioning	Instantaneous total effect	Instantaneous natural direct effect	Instantaneous natural indirect effect	Relative instantaneous natural indirect effect
no	1	931.5	931.4	0.1	<0.001
no	2	235.8	232.8	3.0	0.013
no	3	126.3	103.5	22.8	0.181
no	4	154.4	58.2	96.1	0.623

^a^There was no mediation for the diabetes = yes case

Table 4 contains bias-corrected bootstrapped $$ 95\% $$ CIs for associated estimated instantaneous natural indirect effects of family functioning on child adaptation for the grid of family functioning values 1, 2, 3, and 4, also just for families having children with chronic conditions other than diabetes. Values for the lower and upper bounds increase with family functioning. Compared to Table 2, widths of the CIs are wider in absolute value for the highest value for family functioning (4) and narrower in absolute value otherwise (1–$$ 3) $$. However, the relative width $$ W $$ is larger, $$ 0.84 $$ versus $$ 0.72. $$ In contrast, the associated linear model (i.e., with powers *p* = *q* = 1 and *p*'(0) = *q*'(0) = 1, $$ \rho =0 $$, and having Crohn’s disease or a bowel disorder covariate effects on the means and variances for submodel (14)) has estimated constant instantaneous natural indirect effect $$ 5.5 $$ with bias-corrected bootstrapped $$ 95\% $$ CI $$ 1.4 $$ – $$ 11.7 $$ and normalized width $$ W $$ $$ 0.88 $$. Hence, the moderated monotonic mediation model generates more precise bootstrapped CIs than the standard moderated linear mediation model.

**Table 4:** Bias-corrected bootstrapped $95\%$ confidence intervals for the monotonic mediation model for child adaptation as a function of family functioning as mediated by difficulty controlling for having Crohn’s disease or a bowel disorder and as moderated by having diabetes with independent omitted factors or errors

Diabetes^a^	Family functioning x	Lower bound $L(x)$ on natural indirect effect^b^	Upper bound $U(x)$ on natural indirect effect^b^	Normalized width W of the confidence interval^c^
no	1	0.029	0.18	0.84
no	2	0.93	5.72	0.84
no	3	7.09	43.43	0.84
no	4	29.88	183.00	0.84

^a^There was no mediation for the diabetes = yes case

^b^Using 1,000 resamples

^c^ *W* = (*U*(*x*) − *L*(*x*))/*U*(*x*), which is constant in $x$

In summary, moderated mediation for the family management data is distinctly nonlinearly monotonic. However, mediation only occurs for mothers of children with a chronic condition other than diabetes and not for mothers of children with diabetes.

### Example analyses of the simulated mediation data

Using the CSH covariance structure with $p= q=1$, the first local maximum in the LCV score occurs at $k=15$ with LCV(1,1) $=0.41542$, and so $k=15$ folds are used to compute subsequent LCV scores for the simulated data. The generated CSH model allowing for arbitrary $p$ and $q$ has $p=0$ and $q=0.3$ with distinctly improved $\mathrm{L}\mathrm{C}\mathrm{V}\left(0,0.3\right)=0.93577$ (i.e., the percent decrease for the $p= q=1$ model is 55.6%, much larger than the cutoff of 0.95% for the data). Also, the estimated correlation for this model is the substantial value $0.92$, suggesting highly dependent omitted factors or errors ${U}_M$ and ${U}_Y.$ However, the generated model allowing for arbitrary $p$ and $q$ with uncorrelated omitted factors or errors (i.e.., with $\rho =0$), has $p=0.4$ and $q=1$ (i.e., the true values for these powers) with distinctly improved LCV(0.4,1) $=1.08555$ (i.e., the percent decrease for the model with $p=0$ and $q=0.3$ is 13.8%), indicating that the omitted factors or errors are reasonably treated as independent as simulated. Under this model, *p*' = 1.1 and *q*' = 1.2, close to their simulated values of *p*' = *q*' = 1.

The constant instantaneous indirect effect model associated with this latter model (i.e., with *q*′ = 1) has LCV(0.4,1) $=1.08074$ and insubstantial percent decrease $0.44\%$. Consequently, the instantaneous natural indirect effect is reasonably considered to be constant as simulated. Also, the true model as simulated (i.e., also setting ${p}^{\hbox{'}}=1\Big)$, has LCV(0.4,1) $=1.07928$ and insubstantial percent decrease $0.58\%$, indicating that the true model is a competitive alternative for the simulated data.

Using the constant instantaneous natural indirect effect model (i.e., with $p=0.4$, $q=1$, *p*' = 1.1, and *q*' = 1), the $95\%$ bootstrapped CI with bias correction for that effect is $0.0358$ – $0.0563$, and so contains the true constant instantaneous natural indirect effect $0.04$. The associated $95\%$ bootstrapped CI without bias correction is $0.0363$ – $0.0567$, and so is quite similar, suggesting that bias correction has not inflated the Type I error in this case.

Using the standard linear mediation model (i.e., with $p=1$, $q=1$, *p*' = 1, *q*' = 1, and $\rho =0$), the $95\%$ bootstrapped CI with bias correction for the constant instantaneous indirect effect is $0.0480$ – $0.0974$, and so does not contain the true constant instantaneous natural indirect effect $0.04$. The associated $95\%$ bootstrapped CI without bias correction is $0.0481$ – $0.0986$, and so is quite similar and also does not contain the true value.

## Discussion

We formulated and demonstrated an approach for conducting possibly moderated monotonic mediation analyses based on adaptively selected fractional polynomial models. This formulation considers transformation of outcomes, predictors, and mediators, not just predictors and mediators as previously considered [29–32]. Results of the example analyses of the family management data indicated that transformation of positive valued continuous outcomes can provide distinct improvements over leaving those outcomes untransformed and can resolve problems with model assumptions.

Other nonlinear regression methods could have been used instead to estimate relationships. An advantage of fractional polynomial models is that they are based on linear regression models and so have no more limitations, assumptions, and requirements than models used in linear mediation analyses. Associated derivatives are also readily computed as needed for estimating monotonic instantaneous natural indirect, natural direct, and total effects.

The example analyses used likelihood cross-validation (LCV) for evaluating models, both unadjusted and adjusted for transformation of the outcome. By assessing how a model performs on randomly selected subsets, model evaluation using LCV scores is robust to effects of chance variation compared to using likelihoods based on the complete data. LCV ratio tests, generalizing likelihood ratio tests, can be used to assess whether mediation relationships are constant, linear, or nonlinear as well as a variety of other issues.

The example analyses of the family management data demonstrated the need to address nonlinearity in the context of mediation. Relationships considered in mediation analyses can be distinctly nonlinear. Even when mediation relationships are reasonably treated as linear, consideration of nonlinear alternatives is needed to determine that this assumption holds. It is conventional to treat mediation relationships as linear without checking this assumption. However, like any assumption, it should be checked.

While the example analyses of the family management data demonstrated that distinct nonlinear monotonic mediation can be identified using composite model (12), linear mediation also held. However, the normality assumption was questionable for the linear mediation case and also the constant variances assumption. There are likely to be data sets where mediation can be identified only by consideration of monotonicity. However, there are also likely to be data sets where consideration of monotonicity does not resolve problems related to the normality and constant variances assumptions. Quantile regression methods [26] can be used in such cases.

The example data analyses of the family management data also demonstrated that standard linear mediation analyses can provide a misleading impression that the natural indirect effect is constant in the predictor $X$ when the indirect effect can actually vary quite a bit from this constant value. Moreover, the single $95\%$ bootstrapped CI generated by the linear moderation analysis can be less precise than the CIs generated by a monotonic mediation analysis and can even not overlap for some values of the predictor $X$, suggesting that a linear approach can generate biased indirect effects. Furthermore, the example analyses of the family management data demonstrated that moderated mediation analyses are important to consider because mediation may be weaker for some subpopulations than others and even not hold for some subpopulations (e.g., mothers of children with diabetes compared to mothers of children with other chronic conditions).

The example analyses of the simulated mediation data support the effectiveness of adaptive mediation modeling since the true model was a competitive alternative to the adaptively selected model and since bootstrapped $95\%$ CIs for the constant instantaneous indirect effect contained the true value. However, these analyses also demonstrate that allowing for correlated omitted factors or errors when in fact they are independent can generate models quite different from the true model, indicating the importance of conducting analyses of both cases. These analyses also demonstrate that conducting a standard linear mediation analysis when in fact the relationship (9) is nonlinear in the outcome (i.e., $p\ne 1$) but when the true instantaneous natural indirect effect is constant (i.e., $q^{\prime }=1$) can result in a biased $95\%$ CI for that constant effect. This is also likely to hold when relationships (8)-(9) are nonlinear in the mediator (i.e., $q\ne 1$).

### Limitations

The example mediation analyses of the family management data were limited since they were based on cross-sectional data, and so the timing of measurements for predictors, mediators, and outcomes could not be controlled to reflect precedence as needed to support causality [11, 44]. These analyses were also limited by the absence of control over the predictor variable given the non-experimental design of the study [21]. However, the primary purpose of those analyses was to demonstrate nonlinear mediation and the need for such analyses. This purpose was effectively achieved by the example analyses. Further work, though, is needed to address monotonic mediation in situations where the timing of measurement of variables has been controlled and where the predictor is experimentally controlled. An advantage of an experimentally controlled predictor is that it would be categorical and hence not require transformation. However, the outcome $Y$ and the mediator $M$ might benefit from transformation. There is also a need to replicate these analyses using a wider variety of data sets.

Nonlinearity was addressed here for mediation involving univariate outcomes. However, mediation is also conducted using repeated measurements, either over clusters or longitudinally over time, analyzed with multilevel modeling, linear mixed modeling, or structural equation modeling [6, 13, 57–61]. However, linear relationships are usually assumed in these analyses. Further research is needed to extend monotonic mediation to these cases. The example analyses used a continuous outcome and mediator, but outcomes and mediators can sometimes be categorical [13]. Further research is needed to extend monotonic mediation to address categorical mediators and/or outcomes. The analyses also only addressed the case with a single mediator. An extension to monotonic mediation is needed that accounts for multiple mediators.

## Conclusions

Mediation relationships are commonly assumed to be linear without assessing the validity of this assumption. Reported example analyses demonstrate that mediation relationships can be nonlinear. Moreover, standard linear mediation analyses can generate models that violate model assumptions and generate biased estimates of indirect effects, but this can in some cases be resolved through more general monotonic mediation analyses. Adaptive methods as extended here to the monotonic mediation and moderated monotonic mediation contexts can effectively account for nonlinearity in mediation relationships. The advantage of restricting to monotonic relationships is that no adjustments are needed to underlying theory about directionality between changes in pairs of variables.

## Abbreviations

CI: Confidence interval
CSH: Compound symmetry heterogeneous
LCV: Likelihood cross-validation
